# Peptide-anchored neutrophil membrane-coated biomimetic nanodrug for targeted treatment of rheumatoid arthritis

**DOI:** 10.1186/s12951-023-01773-x

**Published:** 2023-01-13

**Authors:** Ni Yang, Miaomiao Li, Ling Wu, Yinhong Song, Shi Yu, Yingying Wan, Wenjing Cheng, Baoye Yang, Xiaoqin Mou, Hong Yu, Jing Zheng, Xinzhi Li, Xiang Yu

**Affiliations:** 1 Hubei Key Laboratory of Tumor Microenvironment and Immunotherapy, China Three Gorges University, Yichang, 443002 China; 2 Institute of Infection and Inflammation, China Three Gorges University, Yichang, 443002 China; 3 College of Basic Medical Science, China Three Gorges University, Yichang, 443002 China; 4 The People’s Hospital of China Three Gorges University, Yichang, 443099 China

**Keywords:** Neutrophil, Rheumatoid arthritis, Macrophage, Nanodrug, Target therapy

## Abstract

Macrophage polarization determines the production of cytokines that fuel the initiation and evolution of rheumatoid arthritis (RA). Thus, modulation of macrophage polarization might represent a potential therapeutic strategy for RA. However, coordinated modulation of macrophages in the synovium and synovial fluid has not been achieved thus far. Herein, we develop a biomimetic ApoA-I mimetic peptide-modified neutrophil membrane-wrapped F127 polymer (R4F-NM@F127) for targeted drug delivery during RA treatment. Due to the high expression of adhesion molecules and chemokine receptors on neutrophils, the neutrophil membrane coating can endow the nanocarrier with synovitis-targeting ability, with subsequent recruitment to the synovial fluid under the chemotactic effects of IL-8. Moreover, R4F peptide modification further endows the nanocarrier with the ability to target the SR-B1 receptor, which is highly expressed on macrophages in the synovium and synovial fluid. Long-term in vivo imaging shows that R4F-NM@F127 preferentially accumulates in inflamed joints and is engulfed by macrophages. After loading of the anti-inflammatory drug celastrol (Cel), R4F-NM@F127-Cel shows a significant reduction in hepatotoxicity, and effectively inhibits synovial inflammation and alleviates joint damage by reprogramming macrophage polarization. Thus, our results highlight the potential of the coordinated targeted modulation of macrophages as a promising therapeutic option for the treatment of RA.

**Supplementary Information:**

The online version contains supplementary material available at 10.1186/s12951-023-01773-x.

## Introduction

Rheumatoid arthritis (RA) is a chronic systemic autoimmune disease that is characterized by synovial inflammation and results in synovial hyperplasia and accumulation of synovial fluid through the sustained influx of activated immune cells, such as macrophages, neutrophils, T cells, and B cells, which in turn leads to cartilage, bone destruction, and even loss of joint function [1, 2]. Although the pathogenesis of RA is poorly understood, it is characterized by the infiltration of activated macrophages known as M1-type macrophages, which almost completely cover the synovium of the joint and infiltrate into the synovial fluid of the subsynovial tissue in severe cases [3]. During activation, a large number of cellular pattern recognition receptors, such as the SR-B1 receptor and the Toll-like receptors, are highly expressed on the surface of these macrophages. These cells also release many inflammatory mediators, including TNF-α, IL-1β, IL-6 and metalloproteinases, ultimately leading to synovial hyperplasia and destruction of bone and articular cartilage, indicating that the pathogenesis and severity of RA correlate with the presence of macrophage-derived proinflammatory cytokines within the inflamed synovium and synovial fluid [4]. Meanwhile, in RA, so-called M2-type macrophages secrete IL-10 and play an anti-inflammatory role [5]. Therefore, skewing macrophages toward an anti-inflammatory phenotype is a promising strategy for the treatment of RA.

Emerging evidence suggests that many monomers and active constituents of traditional herbal medicines have potential anti-RA effects through different mechanisms, among which immunoregulation of macrophage polarization has received less attention [6, 7]. In addition, these compounds have faced the problem of low solubility and bioavailability after systemic administration [8]. Moreover, due to the relatively avascular structure of the synovial joint and the limited supply of peripheral blood, the accumulation of anti-RA drugs in target tissues, especially in the synovial fluid, is insufficient, leading to serious systemic adverse reactions and limited therapeutic effects [9]. Therefore, there is a great need to design novel inflammatory tissue-targeted drug delivery vectors to improve drug targeting and bioavailability, reduce systemic toxicity, and prevent the progression of RA. Recent studies have shown that macrophage-targeted delivery of anti-RA drugs based on folate receptors and scavenger receptor-A could improve RA treatment [10–12]. Despite great efforts, this field is still in its infancy, and there is still a lack of novel strategies to control the modulation of macrophages in the synovial fluid for the effective treatment of RA. Although intra-articular drug delivery methods achieve access to the synovial fluid, the need for repeated joint injections limits their utility and enhances the risk of introducing bacteria into the joint space [13, 14].

As inflammatory cells that play an important role in the development of RA, neutrophils migrate to the inflamed site in a regulated multistep process involving several cell adhesion molecules, chemokines, and chemokine receptors [15]. Studies have shown that adhesion molecules, such as LFA-1, L-selectin (CD62L) and Mac-1, are widely expressed in neutrophils and medicate their extravasation to sites of inflammation [16]. IL-8, a member of the CXC chemokine family, also plays an important role in the recruitment of neutrophils into inflamed joints via CXCR2-mediated chemotaxis [17]. Blockade of IL-8 with a neutralizing antibody can ameliorate arthritis and reduce the infiltration of neutrophils into the joints during the early phase of inflammation, further demonstrating that IL-8 acts as a neutrophil chemotactic factor [18]. In RA patients with active disease, neutrophils were the most abundant cell type present in the synovial fluid, where IL-8 was significantly elevated, indicating that the neutrophils are natural carriers for targeted drug delivery to the synovial fluid. Unfortunately, the direct use of neutrophils as drug carriers is unrealistic due to their intrinsic proinflammatory capacity [19]. In recent years, researchers have utilized neutrophil membranes containing key molecules to develop biomimetic drug delivery systems with inflammation-targeting features [20, 21].

**Fig. 1:**
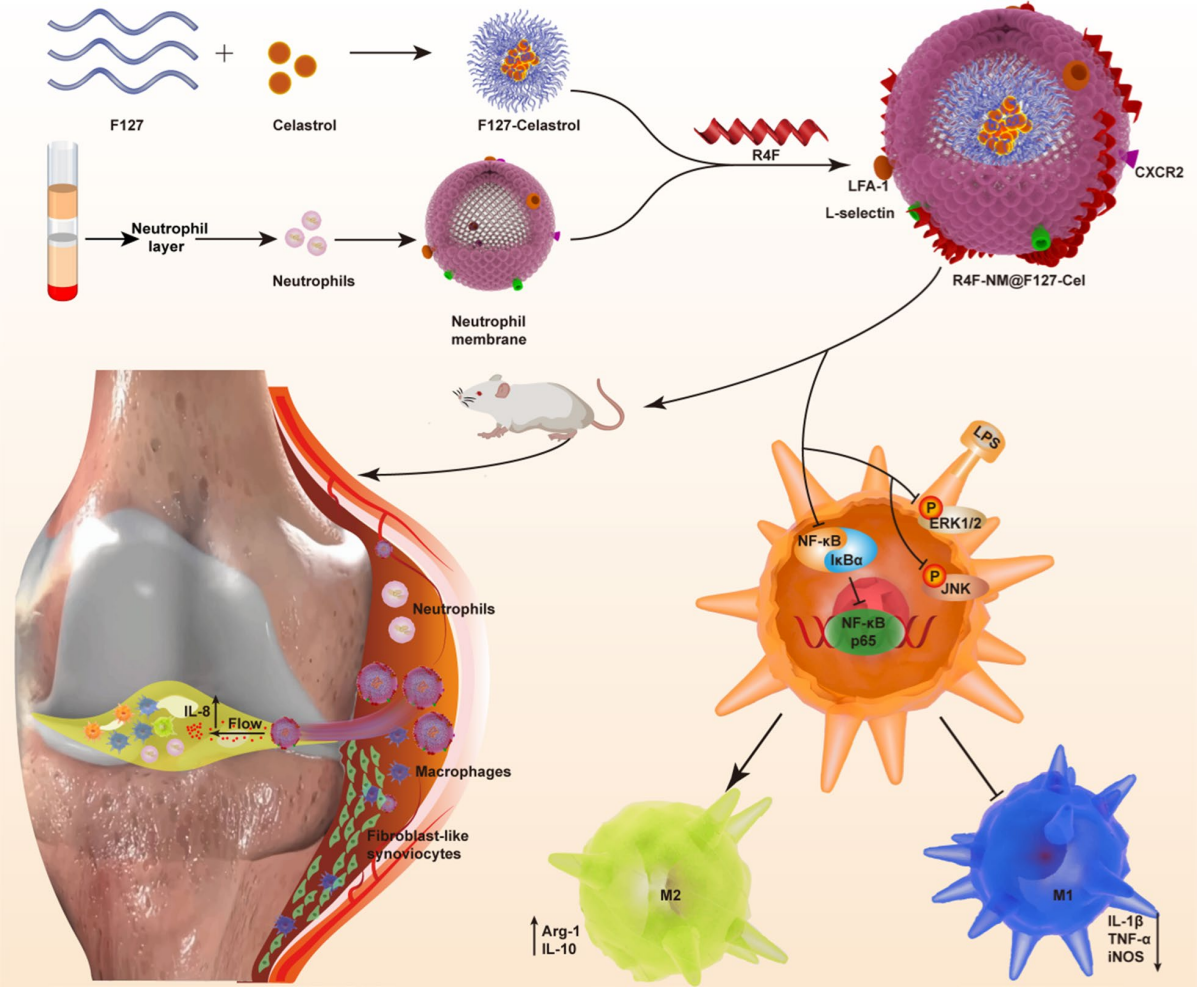
Schematic illustration of in vitro nanoparticle synthesis and in vivo therapeutic mechanisms, including inhibition of synovial inflammation and alleviation of joint damage

Inspired by the intrinsic link between neutrophils and RA, we developed peptide-anchored neutrophil membrane-coated biomimetic nanoparticles (R4F-NM@F127) for targeted drug delivery in RA (Fig. 1). Through coating with neutrophil membranes, nanoparticles disguised as neutrophils could inherit neutrophil membrane proteins and related membrane functions, acquiring the ability to be recruited to the inflamed synovial tissue. Moreover, these biomimetic nanoparticles with a small size could diffuse in the hyperplastic synovial tissue and further penetrate into the synovial fluid under the chemotactic functions of IL-8, which is present at high levels in the synovial fluid. Finally, to achieve specific targeting of macrophages, an ApoA-I mimetic peptide (R4F), which is capable of binding to phospholipid bilayers and targeting the SR-B1 receptor, was anchored to the phospholipid bilayer of the neutrophil membrane. By using in vivo fluorescence imaging, we performed biodistribution and metabolic studies to evaluate the inflammatory tissue- and cell-specific targeting ability of this system. After loading of anti-inflammatory drug, celastrol (Cel), R4F-NM@F127-Cel significantly inhibited M1 macrophage polarization and enhanced M2 macrophage polarization in vitro. In a collagen-induced arthritis (CIA) animal model, R4F-NM@F127-Cel reduced the hepatotoxicity of free Cel and exhibited significant therapeutic efficacy for RA by reprogramming macrophage polarization after systemic administration.

## Results and discussion

### Preparation and characterization of R4F-NM@F127

**Fig. 2:**
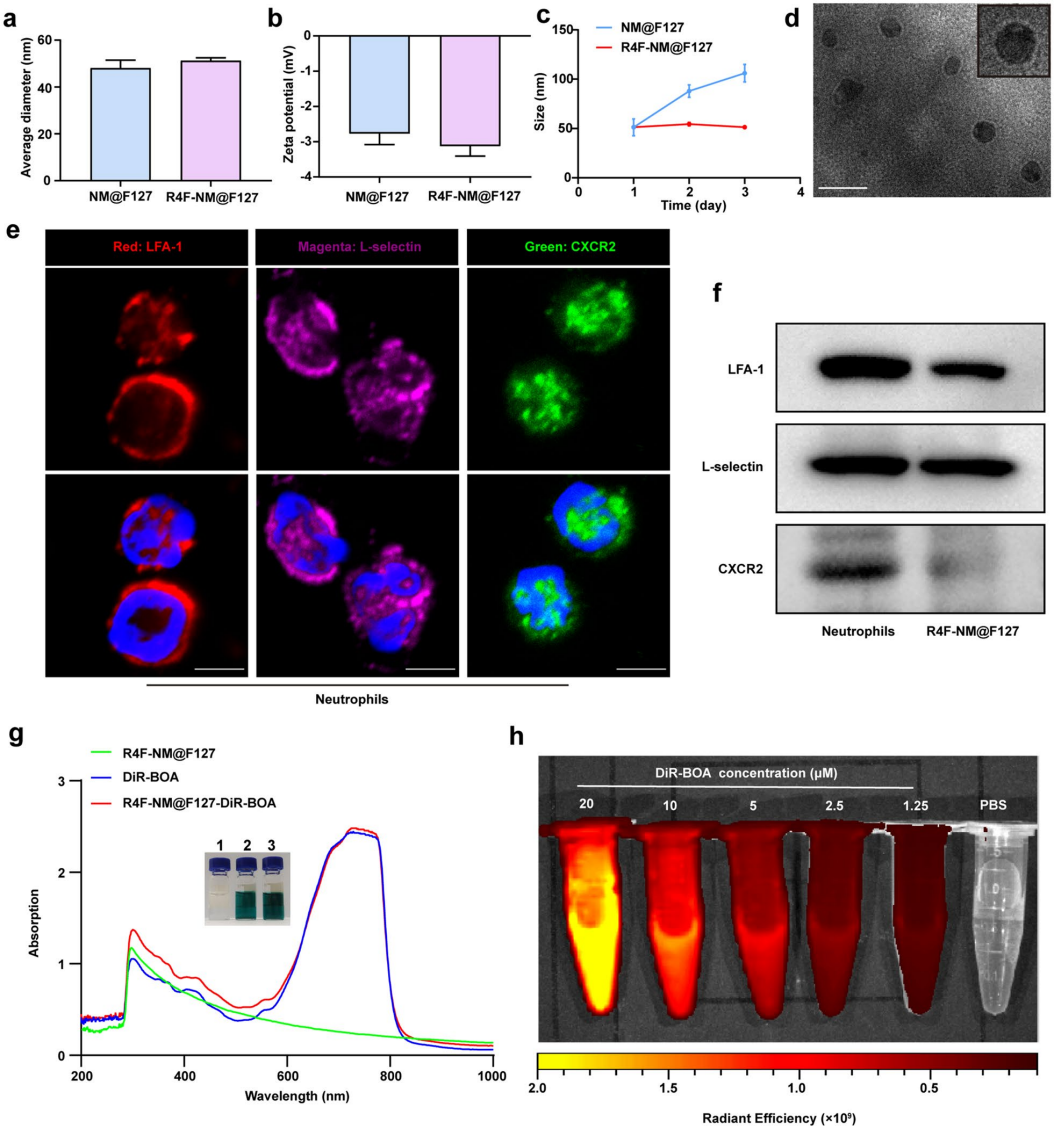
Preparation and characterization of R4F-NM@F127. **a** Average size of NM@F127 and R4F-NM@F127. **b** Zeta potential of NM@F127 and R4F-NM@F127. **c** Stability of NM@F127 and R4F-NM@F127 monitored by DLS. DLS: dynamic light scattering. **d** Representative image of R4F-NM@F127 by TEM. An enlarged image was shown in the upper right. TEM: transmission electron microscopy. Scale bar, 100 nm. **e** LFA-1, L-selectin and CXCR2 on the neutrophil surface were detected by confocal imaging. Scale bar, 5 μm; Blue, DAPI; Red, LFA-1; Magenta, L-selectin; Green, CXCR2. **f** Characteristic protein bands of NMs and R4F-NM@F127 analyzed by western blotting. **g** White light images and UV-Vis absorption spectra of R4F-NM@F127, free DiR-BOA and DiR-BOA-labeled R4F-NM@F127. 1: R4F-NM@F127; 2: free DiR-BOA; 3: DiR-BOA-labeled R4F-NM@F127. **h** A series of images of DiR-BOA-labeled R4F-NM@F127 fluorescence at different concentrations. Data are presented as the mean ± SEM (n = 3)

The synthetic process was divided into three steps. First, lipopolysaccharide (LPS, 1.5 mg/kg) was injected intraperitoneally into the mice to activate neutrophils in vivo. After 6 h, peripheral blood was collected, and neutrophils were isolated with the Mouse Peripheral Blood Neutrophil Isolation Kit (Additional file 1: Fig. S1a). The purity of the neutrophils was more than 95%, and they showed a typical nuclear lobulated morphology after Giemsa staining (Additional file 1: Fig. S1b). Next, to select the optimal extraction method for neutrophil membranes (NMs), we compared the membrane protein content, the number of cell membrane vesicles, the average particle size and the protein expression of lymphocyte function-associated antigen 1 (LFA-1) after three different approaches. The results showed that the NMs obtained by the homogenization method had the highest protein content, the largest number of cell membrane vesicles, the smallest average particle size, and the highest expression of LFA-1 protein expression (Additional file 1: Fig. S2a–d). Therefore, we chose the homogenization method to obtain NMs in subsequent experiments. Second, Pluronic F127 polymer loaded with hydrophobic drugs was prepared by thin-film hydration and was further mixed with NMs to obtain NM@F127 nanoparticles by programmed extrusion. Finally, we functionalized NM@F127 nanoparticles with an ApoA-I mimetic peptide (R4F) that was capable of binding phospholipids on NMs. The average size, zeta potential and polydispersity index (PDI) of NM@F127 and R4F-NM@F127 were determined with a Malvern Zetasizer Nano ZS analyzer. The results showed that the average size of NM@F127 was 48.09 ± 5.82 nm, the zeta potential was − 2.76 ± 0.54 mV, and the PDI was 0.08 ± 0.005. R4F-NM@F127 had values of 51.25 ± 2.086 nm, − 3.12 ± 0.49 mV, and 0.078 ± 0.004, respectively, indicating that the R4F peptide exerted no significant influence on the size, surface charge or PDI of NM@F127 (Fig. 2a, b; Additional file 1: Fig. S3). To investigate the stability of R4F-NM@F127, we continuously monitored the particle size change of R4F-NM@F127 by DLS and found that R4F-NM@F127 was more stable than NM@F127 (Fig. 2c). Transmission electron microscopy (TEM) visualization showed that the obtained R4F-NM@F127 displayed a core-shell structure after negative staining with phosphotungstic acid (Fig. 2d). Moreover, immunofluorescence imaging showed that LFA-1, L-selectin and CXCR2 could be expressed on the surface of neutrophils (Fig. 2e). Western blotting also confirmed the presence and enrichment of these key surface proteins on R4F-NM@F127, further confirming the translocation of NMs onto the F127 polymeric core (Fig. 2f). To verify the drug-loading capacity of R4F-NM@F127, a near-infrared dye (DiR-BOA) was chosen as a lipid-soluble model drug for detection. The white light image showed a distinct color change of R4F-NM@F127 from colorless to black‒blue after encapsulation of DiR-BOA, and the UV–visible absorption spectrum also showed that both free DiR-BOA and DiR-BOA-labeled R4F-NM@F127 had obvious enhancement peaks at 730 nm, whereas the characteristic absorption peaks could not be observed in the absorption spectrum of R4F-NM@F127, indicating the excellent drug encapsulation properties of R4F-NM@F127 (Fig. 2g). In addition, the fluorescence imaging data showed that compared with PBS, DiR-BOA-labeled R4F-NM@F127 had corresponding strong fluorescence signals that increased with increasing concentrations (Fig. 2h).

### The SR-B1 receptor targeting ability of R4F-NM@F127

**Fig. 3:**
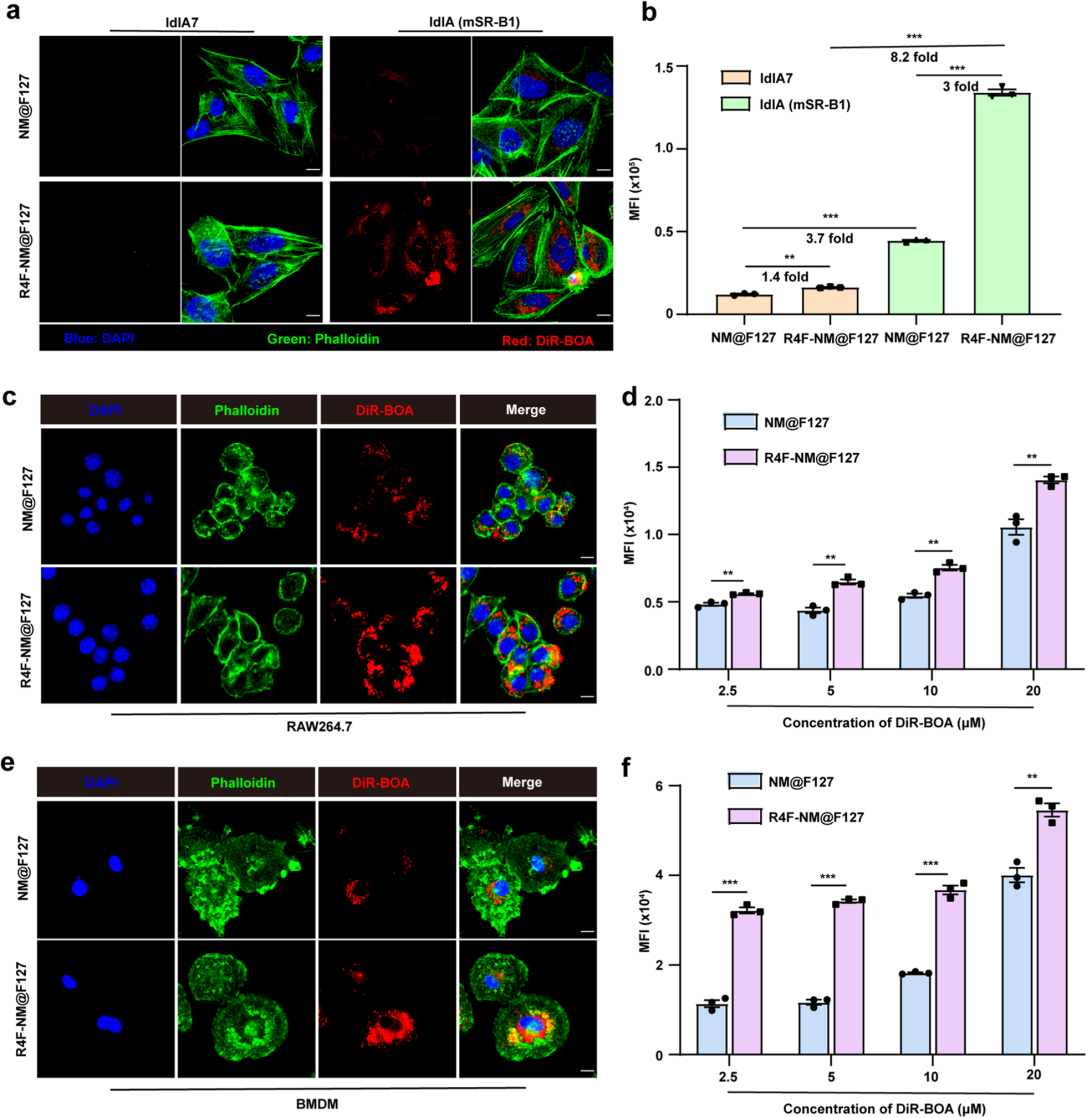
The SR-B1 receptor targeting ability of R4F-NM@F127. **a** Confocal imaging of the SR-B1 targeting ability of R4F-NM@F127 (20 µM). **b** Quantitative analysis of the MFI of DiR-BOA in ldlA7 cells and ldlA (mSR-B1) cells after incubation with DiR-BOA-labeled NM@F127 and R4F-NM@F127. **c** Confocal imaging of the ability of DiR-BOA-labeled R4F-NM@F127 to target RAW264.7 cells in vitro (20 µM). **d** Quantitative analysis of the MFI of DiR-BOA in RAW264.7 cells after incubation with DiR-BOA-labeled NM@F127 and R4F-NM@F127 at different concentrations (n = 3 for each group). **e** Confocal imaging of the ability of DiR-BOA-labeled R4F-NM@F127 to target BMDMs in vitro (20 µM). **f** Quantitative analysis of the MFI of DiR-BOA in BMDMs after incubation with DiR-BOA-labeled NM@F127 and R4F-NM@F127 at different concentrations (n = 3 for each group). Scale bar, 10 μm; Blue, DAPI; Green, phalloidin; Red, DiR-BOA; BMDMs, Bone marrow-derived macrophages; MFI: mean fluorescence intensity; Incubation time: 3 h. Data are shown as the mean ± SEM. **p < 0.01, and ***p < 0.001, as analyzed by unpaired t test

Since the ApoA-I peptide has a high affinity for the SR-B1 receptor, we next used wild-type ldlA7 cells expressing low levels of endogenous SR-B1 and ldlA cells expressing high levels of SR-B1 (mSR-B1) to detect the ability of R4F-NM@F127 to target SR-B1. As shown in Fig. 3a, confocal imaging showed that the uptake capacity of DiR-BOA-labeled R4F-NM@F127 in ldlA (mSR-B1) cells was significantly higher than that in ldlA7 cells. Flow cytometry (FCM) data also showed that the MFI of DiR-BOA labeled R4F-NM@F127 in ldlA (mSR-B1) cells was 8.2-fold greater than that in ldlA7 cells. In addition, the uptake of R4F-NM@F127 by ldlA (mSR-B1) cells was 3-fold greater than the uptake of the untargeted NM@F127 (Fig. 3b). These results suggested that R4F-NM@F127 had a promising SR-B1 receptor-targeting potential. Furthermore, SR-B1 has been well-described as a lipoprotein receptor that is highly expressed on macrophages [22]. To investigate the ability of R4F-NM@F127 to target macrophages, we first incubated two types of macrophages (RAW264.7 cells and BMDMs) with DiR-BOA-labeled NM@F127 and R4F-NM@F127, and the DiR-BOA fluorescence signal intensity in macrophages was analyzed by confocal microscopy and FCM. Confocal imaging results showed that the fluorescence intensity of DiR-BOA in RAW264.7 cells and BMDMs treated with R4F-NM@F127 was significantly stronger than that in NM@F127-treated cells (Fig. 3c, e). Meanwhile, the FCM data showed that RAW264.7 cells and BMDMs took up R4F-NM@F127 in a concentration-dependent manner, with stronger uptake than that observed in the NM@F127 group (Fig. 3d, f). Interestingly, the uptake of R4F-NM@F127 by BMDMs was stronger than that by RAW264.7 cells at the same concentration, possibly due to the overexpression of SR-B1 in BMDMs as primary cell model. Next, the RAW264.7 cells were incubated with R4F-NM@F127 at a DiR-BOA concentration of 20 µM for 3 h at 4 °C to evaluate the targeting ability of R4F peptide mediated by interaction with SR-B1 receptor on macrophages. Fluorescence images showed that R4F-NM@F127 were not significantly internalized into the RAW264.7 cells at 4 °C. However, after incubation at 37 °C for 3 h, strong fluorescence signals were detected in RAW264.7 cells, demonstrating that cellular uptake of R4F-NM@F127 is receptor-dependent mechanism (Additional file 1: Fig. S4). Taken together, these findings suggested that R4F-NM@F127 displayed the ability to selectively target SR-B1 receptor.

### R4F-NM@F127 significantly accumulates in the inflamed joint and further actively targets the arthritic synovium and synovial fluid macrophages through SR-B1-mediated recognition

**Fig. 4:**
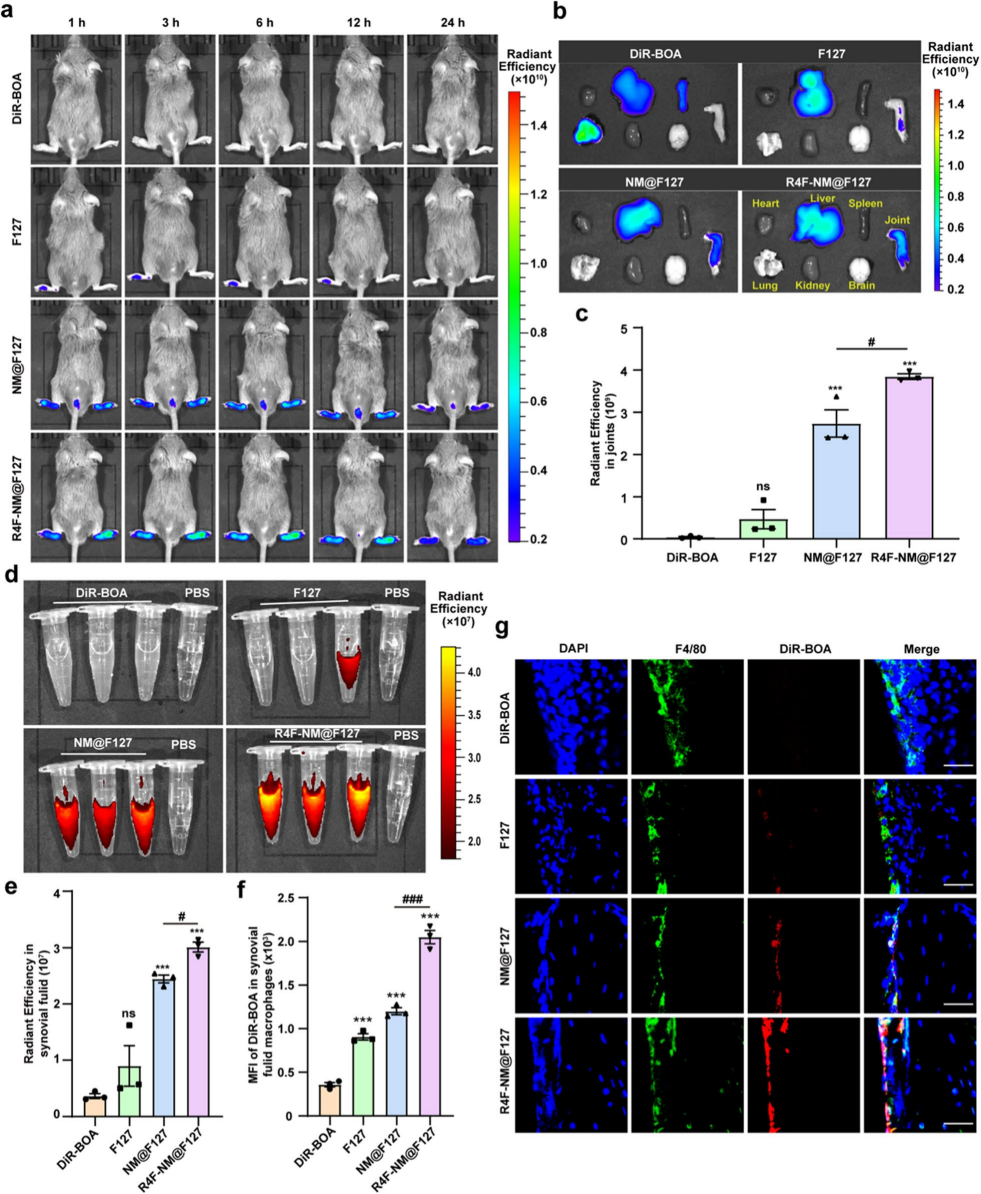
In vivo biodistribution of free DiR-BOA and DiR-BOA-labeled F127, NM@F127 and R4F-NM@F127 in CIA mice. **a** Real-time fluorescence imaging of CIA mice after intravenous injection with DiR-BOA-labeled F127, NM@F127, and R4F-NM@F127 and free DiR-BOA. **b** Ex vivo imaging of the organs at 24 h after intravenous injection. **c** Quantitative analysis of the radiant efficiency in the joints. **d** Fluorescence imaging of synovial fluid collected from CIA mice injected with DiR-BOA-labeled F127, NM@F127, and R4F-NM@F127 and free DiR-BOA. **e** Quantitative analysis of the radiant efficiency in the joint synovial fluid. **f** Quantitative analysis of the MFI of DiR-BOA in synovial fluid macrophages (F4/80^+^CD11b^+^). MFI: mean fluorescence intensity. **g** Fluorescence images of frozen sections of synovial tissue. Scale bar, 50 μm; Blue, DAPI; Green, Synovial macrophages; Red, DiR-BOA. Data are presented as the mean ± SEM (n = 3) and were statistically analyzed using one-way ANOVA followed by Tukey’s multiple comparisons test by comparing the group marked with * with the free DiR-BOA group. ns represents no significant difference; ***p < 0.001; ^#^p < 0.05, and ^###^p < 0.001

To evaluate whether R4F-NM@F127 can be retained in the circulation for a relatively longer time and accumulate preferentially in inflamed joints, DiR-BOA-labeled F127, NM@F127, and R4F-NM@F127 and free DiR-BOA were intravenously administered to CIA mice, and the fluorescence signals of DiR-BOA in the joints were dynamically monitored 1, 3, 6, 12 and 24 h after injection with a live animal imaging system. Compared with those of the free DiR-BOA group, weak fluorescence signals were detected in the legs of arthritic mice injected with DiR-BOA-labeled F127, indicating that only a small amount of DiR-BOA-labeled F127 accumulated at the sites of arthritis by passive targeting. In contrast, injection with both DiR-BOA-labeled NM@F127 and R4F-NM@F127 yielded strong fluorescence signals in the joints of the CIA mice at 1, 3, 6, 12, and 24 h after administration (Fig. 4a), indicating that both NM@F127 and R4F-NM@F127 can accumulate in inflamed joints due to their neutrophil membrane coating. Importantly, the DiR-BOA fluorescence signals in the R4F-NM@F127 group were stronger than those in the NM@F127 group at each time point, suggesting that R4F functional modifications increased the retention of R4F-NM@F127 (Fig. 4a). Following in vivo imaging, the mouse organs and joint tissues were dissected to further assess the arthritis-targeting ability of R4F-NM@F127. Ex vivo imaging also revealed that the DiR-BOA fluorescence signals of NM@F127 and R4F-NM@F127 could be detected in arthritic joints, while the R4F-NM@F127 group had a stronger intensity than NM@F127 (Fig. 4b). Quantitative analysis of the radiant efficiency showed that the CIA mice injected with DiR-BOA-labeled R4F-NM@F127 exhibited a stronger radiant efficiency than did the mice injected with DiR-BOA-labeled NM@F127 at 24 h (Fig. 4c). Although inevitable liver accumulation was observed, the comparable distribution of R4F-NM@F127 in arthritic joints could perfectly compensate its limitation (Additional file 1: Fig. S5). Thus, these results indicated that R4F-NM@F127 and NM@F127 were able to target and accumulate in inflamed joints, but R4F-NM@F127 was more effective due to SR-B1-mediated active targeting. Then, we assessed the distribution of R4F-NM@F127 in the serum by using in vitro serum imaging at 24 h. The imaging data and the quantitative analysis of the DiR-BOA fluorescence intensity showed that the serum from the R4F-NM@F127 and NM@F127 groups had strong fluorescence signals (Additional file 1: Fig. S6a, b). These results further demonstrated that NM@F127 and R4F-NM@F127 showed relatively long blood circulation times.

IL8 is widely present in RA synovial cells and secreted into the synovial fluid. Given that IL-8 is a very important factor for attracting neutrophils, which are the most abundant cells infiltrating into the synovial fluid [23], we suspected that the NM-coated nanoparticles could further penetrate into the synovial fluid via the chemotactic functions of IL-8. Next, we collected the synovial fluid to assess the distribution of R4F-NM@F127. In vitro imaging clearly showed that there were much stronger DiR-BOA fluorescence signals in the synovial fluid from the CIA mice injected with R4F-NM@F127 and NM@F127 than in the synovial fluid from the mice in the free DiR-BOA and F127 groups (Fig. 4d). It is worth noting that the DiR-BOA fluorescence signals in the R4F-NM@F127 groups were stronger than those in the NM@F127 group, indicating that R4F-NM@F127 was arrested in the synovial fluid rather than rapidly flowing out from the joint cavity. The quantitative fluorescence results were consistent with the imaging data (Fig. 4e). In addition, the FCM analysis results showed that the fluorescence intensity of DiR-BOA in synovial fluid macrophages in the R4F-NM@F127 group was stronger than that in the NM@F127 group, indicating that R4F modification can enhance the uptake of NM@F127 by synovial fluid macrophages (Fig. 4f). In addition to synovial fluid macrophages, the macrophages found in the synovial tissue also play a key role in the pathogenesis of RA [24]. To further evaluate the colocalization of R4F-NM@F127 with joint synovial macrophages, the joint synovium was subjected to immunofluorescence staining. Confocal imaging data showed that there were very weak DiR-BOA fluorescence signals in both the free DiR-BOA and F127 groups. However, there was a significantly stronger DiR-BOA fluorescence signal in the joint synovium in the R4F-NM@F127 group than in the NM@F127 group (Fig. 4g). Meanwhile, DiR-BOA-labeled R4F-NM@f127 colocalized well with synovial macrophages. Importantly, we found that NM@f127 also seems to be engulfed by synovial macrophages. Studies have shown that AnxA1 is expressed on the membrane of neutrophils and can interact with its receptor FPR2 (formyl peptide receptor 2), while FPR has been shown to exist in macrophages and hepatocytes [25]. Taken together, NM@F127 and R4F-NM@F127 can accumulate in inflamed joints, but R4F-NM@F127 can further actively target the arthritic synovium and synovial fluid macrophages via SR-B1-mediated recognition.

### R4F-NM@F127-Cel can significantly reprogram RAW264.7 cells polarization by inhibiting the activation of NF-κB and MAPK signaling in vitro

**Fig. 5:**
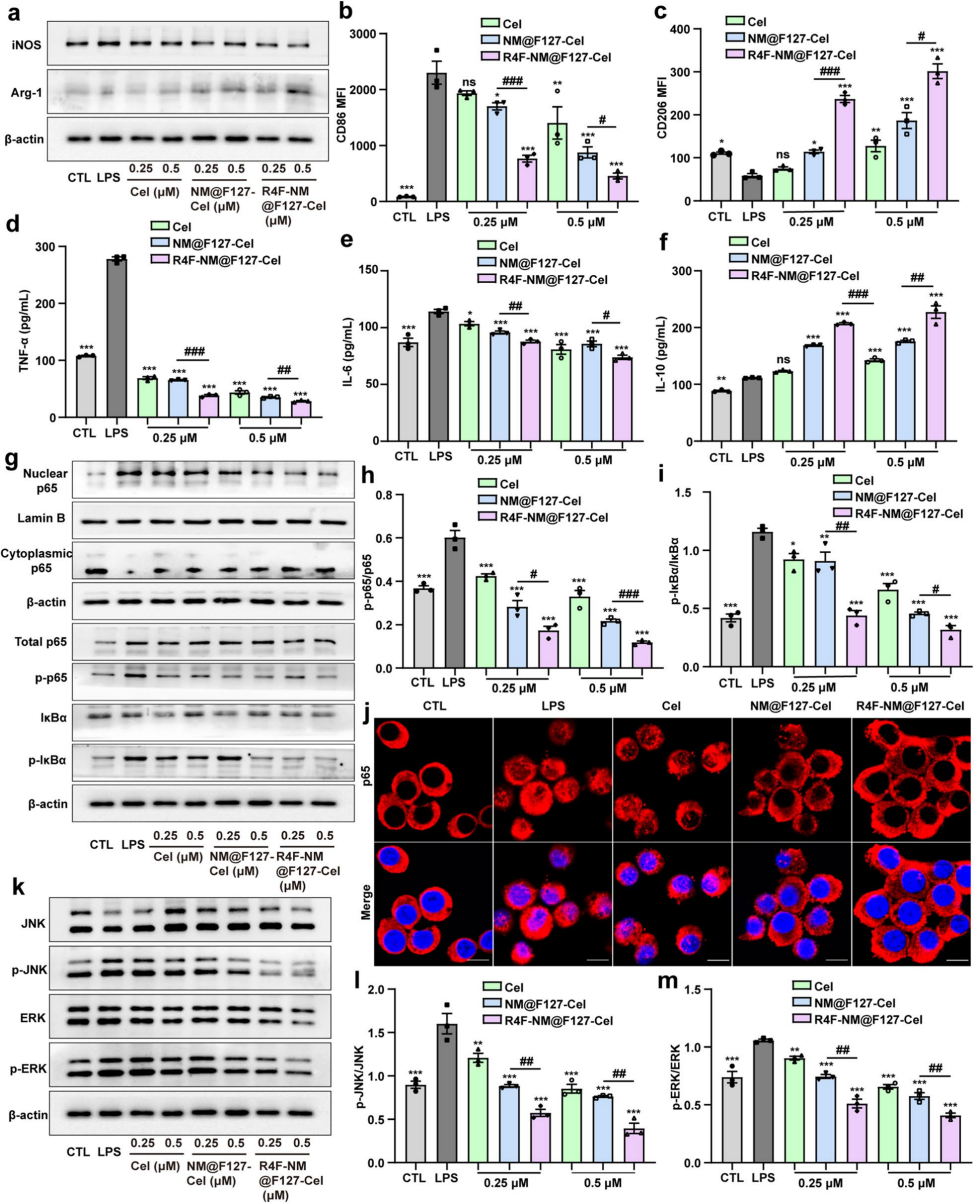
Effects of R4F-NM@F127-Cel on LPS-induced RAW264.7 cells polarization in vitro. **a** iNOS and Arg-1 protein expression in LPS-treated RAW264.7 cells after different treatments, as evaluated by western blot assay. **b–c** MFI of CD86 and CD206 on LPS-treated RAW264.7 cells by flow cytometry. MFI: mean fluorescence intensity. **d–f** ELISA results for TNF-α (**d**), IL-6 (**e**) and IL-10 (**f**) secretion in LPS-treated RAW264.7 cells supernatants from various treatment groups. **g** Effects of different treatments on the expression levels of components of the NF-κB pathway, including total p65, p-p65, IκBα, p-IκBα, cytoplasmic p65 and nuclear p65, as determined by western blotting. **h–i** Quantitative analysis of p-p65/p65 and p-IκBα/IκBα protein levels. **j** Confocal fluorescence images of p65 nuclear localization. Scale bar, 5 μm; Blue, DAPI; Red, p65. **k** Effects of different treatments on the expression levels of compontents of MAPK pathway, including JNK, p-JNK, ERK and p-ERK, as determined by western blotting. **l–m** Quantitative analysis of p-JNK/JNK and p-ERK/ERK protein levels. Data are presented as the mean ± SEM (n = 3) and were statistically analyzed using one-way ANOVA followed by Tukey’s multiple comparisons test by comparing the group marked with * with the LPS group. ns represents no significant difference. *p < 0.05, **p < 0.01, ***p < 0.001; ^#^p < 0.05, ^##^p < 0.01 and ^###^p < 0.001

M1 macrophages are dominant in the RA synovium and synovial fluid, and release many inflammatory cytokines that contribute to cartilage and bone destruction [26]. Next, we chose Cel as an anti-inflammatory drug and evaluated the anti-inflammatory effect of R4F-NM@F127-Cel in vitro. The characterization results showed that the average diameter and charge of R4F-NM@F127-Cel and NM@F127-Cel were almost the same as before (Additional file 1: Fig. S7). The drug loading of R4F-NM@F127-Cel was detected by high-performance liquid chromatography (HPLC). The UV–visible absorption spectrum showed that both free Cel and R4F-NM@F127-Cel had obvious enhancement peaks at 415 nm, and the white light image also showed a distinct color change of R4F-NM@F127 from colorless to yellow after encapsulation of Cel (Additional file 1: Fig. S8). Subsequently, we determined the expression levels of the typical M1/M2 macrophage biomarkers by RT‒qPCR. Compared to those in the control (CTL) groups, the mRNA expression levels of the M1 macrophage markers iNOS, TNF-α and IL-1β were significantly increased in RAW264.7 cells activated with LPS, while the levels of the M2 macrophage markers IL-10 and Arg-1 were decreased (Additional file 1: Fig. S9a–e). In addition, R4F-NM@F127-Cel markedly inhibited the mRNA expression of iNOS, TNF-α, and IL-1β and increased the mRNA expression of IL-10 and Arg-1 in a concentration-dependent manner. Free Cel and NM@F127-Cel also inhibited the mRNA expression of iNOS, TNF-α, and IL-1β while increasing IL-10 and Arg-1 mRNA expression, but they had a weaker effect than R4F-NM@F127-Cel (Additional file 1: Fig. S9a–e). Next, the effect of R4F-NM@F127-Cel on RAW264.7 cells polarization was further detected by western blotting and FCM. The western blot results showed that the expression of iNOS was significantly upregulated and the expression of Arg-1 was downregulated in the cells following LPS activation. However, the three treatment groups (free Cel, NM@F127-Cel and R4F-NM@F127-Cel) showed downregulation of iNOS expression and upregulation of Arg-1 expression in a concentration-dependent manner, and the effect of R4F-NM@F127-Cel was more obvious (Fig. 5a). Moreover, quantitative analysis further confirmed that R4F-NM@F127-Cel treatment had a stronger effect, downregulating the expression of iNOS and upregulating the expression of Arg-1 (Additional file 1: Fig. S10). Meanwhile, the changes in the levels of the M1 macrophage marker CD86 and the M2 macrophage marker CD206 were further detected by FCM. The MFI values of CD86 and CD206 showed a pattern similar to that observed in western blotting; that is, R4F-NM@F127-Cel treatment could downregulate the expression of CD86 and upregulate the expression of CD206 (Fig. 5b, c). The anti-inflammatory activity of R4F-NM@F127-Cel was further assessed by measuring the expression of TNF-α, IL-6, and IL-10, three important pro- and anti-inflammatory cytokines expressed after LPS-induced RAW264.7 cells polarization. The enzyme-linked immunosorbent assay (ELISA) results demonstrated that R4F-NM@F127-Cel reduced TNF-α and IL-6 secretion while increasing IL-10 secretion (Fig. 5d–f). These results indicate that R4F-NM@F127-Cel can significantly inhibit the M1 polarization of RAW264.7 cells and induce M2 polarization in a concentration-dependent manner by actively targeting of macrophages.

When NF-κB signaling in macrophages is abnormally activated, it can promote autoimmunity and inflammation, thus exacerbating RA synovitis and bone erosion [27]. Previous studies have shown that Cel can downregulate NF-κB signaling [28]. Next, the expression levels of the critical proteins related to the NF-κB signaling pathway were detected by western blotting. As shown in Fig. 5g, LPS stimulation dramatically activated the phosphorylation of p65 and IκBα, and promoted cytoplasmic p65 entry into the nucleus. However, the three treatment groups (free Cel, NM@F127-Cel and R4F-NM@F127-Cel) showed attenuated LPS-induced phosphorylation of p65 and IκBα in a concentration-dependent manner, as well as inhibition of cytoplasmic p65 entry into the nucleus. Quantitative analysis further confirmed that the R4F-NM@F127-Cel treatment inhibited the phosphorylation of p65 and IκBα more than the free Cel and NM@F127-Cel treatments. Meanwhile, R4F-NM@F127-Cel treatment significantly inhibited the nucleation of cytoplasmic p65 (Fig. 5h, i; Additional file 1: Fig. S11). We also assessed the effect of R4F-NM@F127-Cel on the nuclear translocation of the NF-κB p65 subunit by immunofluorescence staining. The results showed that LPS-induced p65 translocation to the nucleus was more strongly inhibited by R4F-NM@F127-Cel than by free Cel and NM@F127-Cel (Fig. 5j). In addition, the mitogen-activated protein-kinases (MAPKs) are a group of highly conserved mitin-activated protein kinases that play important roles in regulating the inflammatory response and cellular stress [29]. We hypothesize that R4F-NM@F127-Cel exerts potent anti-inflammatory effects through actively targeted inhibition of the MAPK signaling pathway. The results revealed that LPS activated the phosphorylation of ERK and JNK in the MAPK signaling pathway in LPS-stimulated RAW264.7 cells (Fig. 5k). However, quantitative analysis showed that compared to the free Cel and NM@F127-Cel treatments, R4F-NM@F127-Cel treatment significantly reduced the phosphorylation of ERK and JNK in a concentration-dependent manner (Fig. 5l, m). Collectively, these results indicated that R4F-NM@F127-Cel could markedly inhibit the inflammatory response by suppressing the activation of NF-κB and MAPK signaling.

### R4F-NM@F127-Cel can significantly inhibit synovial inflammation and alleviate joint damage in CIA mice

**Fig. 6:**
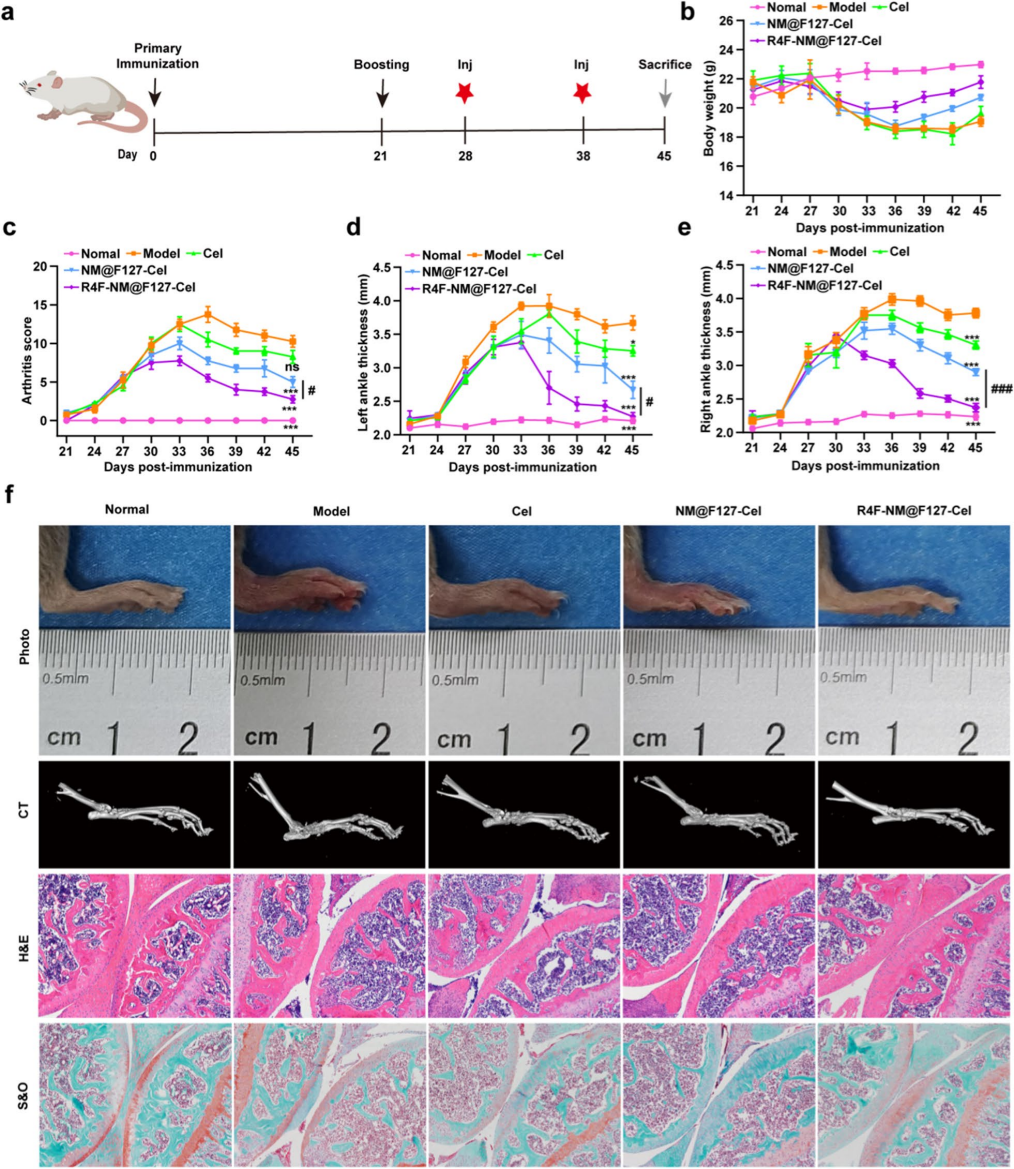
In vivo therapeutic effects of free Cel, NM@F127-Cel and R4F-NM@F127-Cel in CIA mice. **a** Experimental schedule of CIA induction and R4F-NM@F127-Cel injection. Inj, injection. **b–e** CIA clinical symptoms were effectively attenuated by intravenous administration of different treatments. The body weight (**b**), clinical scores (**c**), and ankle diameter (mm) of the left hind paw (**d**) and the right hind paw (**e**) of mice were evaluated every 3 days until sacrifice. **f** Representative gross lesions, micro-CT examination, H&E staining, and S&O staining. (100×). Data are presented as the mean ± SEM (n = 4) and were statistically analyzed using one-way ANOVA followed by Tukey’s multiple comparisons test by comparing the group marked with * with the model group. ns represents no significant difference. *p < 0.05, ***p < 0.001; ^#^p < 0.05, and ^###^p < 0.001

To further evaluate the therapeutic effect of R4F-NM@F127-Cel on RA, the different Cel-loaded nanoparticles were administered to CIA mice at a Cel dose at 5 mg/kg body weight every 10 days by intravenous injection starting on day 28 of primary immunization after arthritis induction (Fig. 6a). Subsequently, the body weight, arthritis scores, and paw thickness of the left and right sides were measured every 3 days from day 21 after the first immunization. Body weight serves as an indirect indicator of therapeutic efficacy in RA. As shown in Fig. 6b, the body weight in the mice injected with R4F-NM@F127-Cel increased continuously during the latter period of treatment and almost reached the values observed in the normal group. Furthermore, changes in arthritis scores are also a crucial indicator to assess anti-inflammatory effects during RA progression. Compared to the mice in the model group treated with PBS, the mice treated with free Cel, NM@F127-Cel, or R4F-NM@F127-Cel had significantly lower arthritis scores. Notably, the R4F-NM@F127-Cel-treated group had the lowest arthritis scores (Fig. 6c; Additional file 1: Fig. S12a). Next, the paw thickness on the left and right sides was measured in CIA mice treated as described before. Similarly, CIA mice treated with free Cel, NM@F127-Cel, and R4F-NM@F127-Cel showed obvious improvements in paw thickness (Fig. 6d, e; Additional file 1: Fig. S12b, c). Notably, the paw thickness in the R4F-NM@F127-Cel-treated group was more significantly improved than that in the NM@F127-Cel- and free Cel-treated groups, which was consistent with the arthritis scores (Fig. 6d, e; Additional file 1: Fig. S12b, c). Thus, these results clearly demonstrated that the R4F-NM@F127-Cel markedly improved clinical outcomes, including body weight, arthritis scores, and the paw thickness.

Imaging of the left hind limbs in the model group treated with PBS showed severe, extensive joint swelling and joint deformation, while free Cel reduced these symptoms slightly but not markedly (Fig. 6f). Remarkably, R4F-NM@F127-Cel reduced this swelling nearly completely, such that the difference from the normal group was not significant. Although NM@F127-Cel also significantly ameliorated swelling, the effect was obviously not as effective as that of R4F-NM@F127-Cel. Bone erosion and destruction are major pathological features of rheumatoid arthritis and are often used to monitor the severity of the disease [30]. Micro-CT analyses were used to further investigate bone changes in different treatment groups. The model group showed the most severe damage, with extensive erosion of the bone in the ankle and toe joints. However, R4F-NM@F127-Cel was associated with significantly lower ankle bone erosion than NM@F127-Cel, resulting in bones that were similar to those in normal mice, indicating that R4F modification further improves the anti-RA effect of NM@F127-Cel (Fig. 6f). To further confirm the efficacy of targeted nanoparticle treatment in CIA mice, we performed histological analysis with H&E and saffron O staining for joints from different treatment groups. The results showed no signs of inflammation and no cartilage destruction in the normal group. In addition, the interface between bone and cartilage could be clearly distinguished by their morphology (Fig. 6f; Additional file 1: Fig. S13). However, the model group of PBS-treated CIA mice showed serious inflammatory cell infiltration and cartilage destruction, and it was difficult to identify the intact articular cavity. In addition, the interface between bone and cartilage was hard to distinguish due to the infiltration of inflammatory cells as well as the erosion of cartilage and bone. In both the NM@F127-Cel group and the R4F-NM@F127-Cel group, synovial inflammation and cartilage erosion were significantly reduced, but the R4F-NM@F127-Cel group had an obviously better effect. Meanwhile, the histomorphology in the R4F-NM@F127-Cel group was nearly identical to that in the normal group, indicating that R4F-NM@F127-Cel exhibits satisfactory therapeutic efficacy for RA therapy (Fig. 6f; Additional file 1: Fig. S13). Accordingly, compared to that of free Cel or NM@F127-Cel at the same dose, the therapeutic benefit of R4F-NM@F127-Cel on RA was clearly demonstrated. Altogether, these results suggest that R4F-NM@F127-Cel can significantly inhibit synovial inflammation and alleviate joint damage in CIA mice.

### R4F-NM@F127-Cel can significantly reprogram macrophage polarization in the joint synovium and synovial fluid of CIA mice to alleviate joint damage

**Fig. 7:**
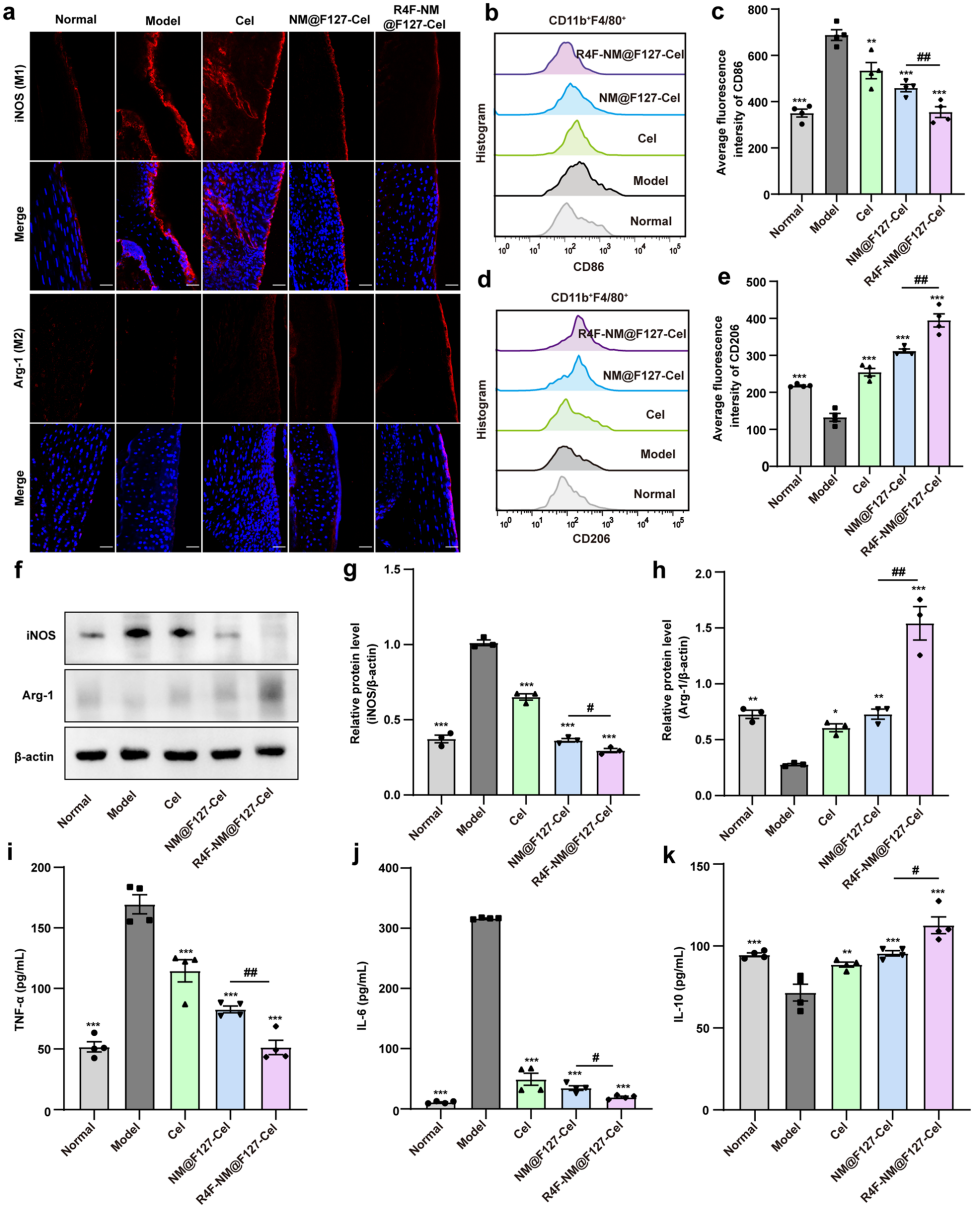
In vivo anti-inflammatory mechanism of free Cel, NM@F127-Cel and R4F-NM@F127-Cel in CIA mice. **a** Confocal imaging of frozen sections of synovial tissue in joints stained with the M1 macrophage marker (iNOS) and M2 macrophage marker (Arg-1). Scale bar, 50 μm; Blue, DAPI; Red, Synovial macrophages. **b–e** Average fluorescence intensities and statistical results of M1 and M2 macrophage populations in synovial fluid from CIA mice from different groups analyzed by FCM (n = 4 for each group). FCM: flow cytometry. **f** The expression of iNOS and Arg-1 in joint synovial tissue from CIA mice was analyzed by western blotting. β-actin was used as a loading control. **g–h** Quantitative analysis of iNOS and Arg-1 protein levels relative to β-actin (n = 3 for each group). **i–k** Serum levels of cytokines (TNF-α, IL-6 and IL-10) after different treatments (n = 4). Data are presented as the mean ± SEM and were statistically analyzed using one-way ANOVA followed by Tukey’s multiple comparisons test by comparing the group marked with * with the model group. *p < 0.05, **p < 0.01, ***p < 0.001; ^#^p < 0.05, ^##^p < 0.01

To further understand of the mechanisms underlying the therapeutic effects we observed, the effects of R4F-NM@F127-Cel on the macrophage polarization in vivo were evaluated by detecting the expression of specific phenotypic markers of M1 and M2 macrophages in arthritic joint synovium tissues from CIA mice. As shown in Fig. 7a, compared with that of the normal group, iNOS expression was markedly increased in the arthritic joint synovial tissues of the model group. Meanwhile, the expression levels of iNOS were decreased to varying degrees after treatment in the free Cel group and NM@F127-Cel group, accompanied by an increase in Arg-1 levels. More importantly, R4F-NM@F127-Cel treatment prominently decreased iNOS expression and increased Arg-1 expression, implying that R4F-NM@F127-Cel can significantly inhibit M1 polarization in joint synovial tissue and induce M2 polarization. In addition, FCM analysis of phenotypic changes in the synovial fluid macrophage subpopulation was further explored. The average fluorescence intensities of the M1 macrophage-specific biomarker CD86 decreased and those of the M2 macrophage-specific biomarker CD206 increased obviously in the R4F-NM@F127-Cel treatment group compared with the NM@F127-Cel group (Fig. 7b–e). These results were consistent with the results of the immunofluorescence analyses. As expected, the R4F-NM@F127-Cel treatment group exhibited significantly reduced M1 polarization and increased M2 phenotypic polarization in the arthritic joint synovial fluid, which contributed to optimal anti-inflammatory efficacy in vivo. Next, we further examined the polarization of macrophages in the joint synovial tissue of CIA mice by western blotting. The results showed that the expression of iNOS was obviously increased, and the expression of Arg-1 was decreased in the model group compared with the normal group. In contrast, the three treatment groups (free Cel, NM@F127-Cel and R4F-NM@F127-Cel) showed downregulated iNOS expressions and upregulated Arg-1 expressions (Fig. 7f). Moreover, quantitative analysis confirmed that the R4F-NM@F127-Cel treatment had a greater ability to downregulate iNOS expression and increase Arg-1 expression than Cel and NM@F127-Cel (Fig. 7g, h). These results indicated that the R4F-NM@F127-Cel treatment group significantly inhibited the production of proinflammatory M1 macrophages and promoted the polarization of anti-inflammatory M2 macrophages in the synovium and synovial fluid. Next, ELISA was carried out to examine the cytokine expression profiles (TNF-α, IL-6 and IL-10) in the serum after different treatments. Compared to the expression levels of these inflammatory cytokines in the normal group, the expression of TNF-α and IL-6 was significantly increased in the model group; however, the expression of IL-10 was decreased (Fig. 7i–k). In comparison with the model group, the free Cel, NM@F127-Cel and R4F-NM@F127-Cel groups showed significantly decreased expression of TNF-α and IL-6 and increased expression of IL-10 (Fig. 7i–k). Overall, R4F-NM@F127-Cel significantly inhibited synovial inflammation and alleviated joint damage by reprogramming macrophage polarization.

A key question for nanoparticle based therapy is whether nanoparticle delivery is toxic to normal tissue. Studies have reported that Cel is effective in the treatment of RA by suppressing inflammatory responses and protecting against bone destruction [8, 31]. However, its anti-arthritic effect has not been fully utilized clinically due to its multiorgan toxicity and poor solubility. Therefore, we dissected major organs at day 45 after the first immunization, and a nanoparticle toxicity assessment was performed. The heart, spleen, lung, kidney, and brain of the mice in all groups appeared smooth and normal in color and size. In addition, histological examinations for these organs indicated a normal physiological structure and cell morphology (Additional file 1: Fig. S14). However, the results of the free Cel treatment group showed that the activities of ALT and AST, which are biochemical markers of liver injury, were significantly increased, and histological liver injury, including infiltration of inflammatory cells, liver cell necrosis, and nuclear fragmentation, was observed (Additional file 1: Figs. S14, S15). This adverse effect of Cel was largely abolished after treatment with the encapsulated R4F-NM@F127 nanoparticles. Therefore, R4F-NM@F127 nanoparticle encapsulation markedly reduces the hepatotoxicity effects and consequently enhances the safety profile of Cel treatment.

## Conclusion

In this study, we exploited a bioinspired ApoA-I mimetic peptide-modified neutrophil membrane-coated nanocarrier (R4F-NM@F127) to achieve targeted anti-RA therapy. Neutrophil membrane coating endowed this carrier with the ability to target the inflamed synovial tissue and enter the synovial fluid via the chemotactic functions of IL-8, which is present at high levels in synovial fluid from patients with RA. More importantly, R4F peptide modification further endowed the nanocarrier with the ability to target SR-B1-positive macrophages in the synovium and synovial fluid, leading to strong inflammatory tissue- and cell-specific accumulation. In the CIA model, Cel-loaded R4F-NM@F127 reduced the hepatotoxicity of free Cel and effectively inhibited synovial inflammation and alleviated joint damage by reprogramming macrophage polarization. Based on pathological changes and pathogenetic mechanisms of RA, we first propose a novel strategy to target the macrophages in the synovial fluid for the effective treatment of RA. Therefore, we believe that the coordinated modulation of macrophages in the synovium and synovial fluid might provide an new perspective for RA therapy.

## Experimental section

### Materials

The DiR-BOA (1,1′-dioctadecyl-3,3,3′,3′-tetramethylindotricarbocyanine iodide bisoleate) was generously provided by Professor Cao of Shanghai University. R4F (an ApoA1-mimetic peptide, Ac-FAEKFKEAVKDYFAKFWD) was synthesized by Shanghai Apeptide Co., Ltd (Shanghai, China). Celastrol was obtained from MedChemExpress (New Jersey, USA). The ldlA7 (SR-B1^−^), ldlA (mSR-B1) (SR-B1^+^) cell lines were gifts from Dr. Monty Krieger (Massachusetts Institute of Technology, Cambridge, MA). ELISA kits for tumor necrosis factor-α (TNF-α), interleukin-6 (IL-6) and interleukin-10 (IL-10) were supplied by MultiSciences Biotech Co., Ltd (Lianke, China). Murine macrophage colony-stimulating factor (M-CSF) was purchased from PeproTech. Protease Inhibitor Cocktail was purchased from Sigma–Aldrich. Fetal bovine serum (FBS), penicillin/streptomycin and Dulbecco’s modified Eagle’s medium (DMEM) were bought from Gibco (USA). Dimethyl sulfoxide (DMSO) was bought from Sigma (USA). OCT compound was purchased from Sakura Finetek (Torrance, CA, USA). Antibodies β-actin was bought from Boster Company. HRP labeled antibody, Giemsa stainand, EGTA, Nuclear and Cytoplasmic Protein Extraction Kit, Anti-fluorescence quenching sealant and 4′,6-diamidino-2-phenylindole (DAPI) were bought from Beyotime Biotechnology Co., Ltd. LFA-1 was purchased Invitrogen. CXCR2, iNOS, Arg-1, JNK, p-JNK, ERK and p-ERK were purchased from Proteintech (USA). Lamin B, p-p65, IκBα and p-IκBα were purchased from Wanleibio Co., Ltd. Goat Anti-Rabbit IgG H&L (Alexa Fluor® 594) and anti-NF-kB p65 antibody were were purchased from Abcam. Antibodies to CD16/32, CD11b, F4/80, CD206, CD86 were purchased from Biolegend (USA). RNA isolater Total RNA Extraction Reagent, HiScript® III RT SuperMix for qPCR (+ gDNA wiper), and Taq Pro Universal SYBR qPCR Master Mix were purchased from Vazyme Biotech Co., Ltd. Polycarbonate nuclepore track-etch membranes were purchased from Whatman. Mouse peripheral blood neutrophil isolation Kit, lowry protein concentration determination kit, and Lipopolysaccharide (LPS) were purchased from Beijing Solarbio Science & Technology Co., Ltd. Immunization Grade Bovine type II collagen (CII), Complete Freund’s adjuvant (CFA), and Incomplete Freund’s adjuvant (IFA) were all obtained from Chondrex, Inc. Aspartate aminotransferase (AST) and alanine aminotransferase (ALT) were purchased from Nanjing Institute of Biological Engineering of China.

### Animals and cells

Specific pathogen-free (SPF) kunming (KM) mice (Female, 6–8 weeks old) were purchased from the Laboratory Animal Center of China Three Gorges University (Yichang, Hubei, China). DBA/1 mice (male, 8 weeks old) were obtained from Beijing Huafukang Biotechnology Co., Ltd (Beijing, China). All animal studies were conducted in compliance with protocols that had been approved by the Ethics Committee of China Three Gorges University and in compliance with the experimental guidelines of the National Institutes of Health on the care and use of animals. The RAW264.7 cell line was obtained from Wuhan Procell Life Technology Co., Ltd. The cells were cultured in DMEM containing 10% FBS and 100 U/mL penicillin‒streptomycin at 37 °C in a humidified 5% CO_2_ atmosphere.

### Isolation and identification of peripheral neutrophils

Lipopolysaccharide (LPS, 1.5 mg/kg) was injected intraperitoneally into the mice to activate neutrophils in vivo. After 6 h, peripheral blood from KM mice was collected in tubes containing the anticoagulant EDTA-2K, and neutrophils were isolated with the Mouse Peripheral Blood Neutrophil Isolation Kit. Then, the isolated neutrophils were resuspended in PBS and stored at − 80 °C for subsequent membrane isolation. The cell suspension was stained with Giemsa to further identify neutrophils, and their morphology was examined under an Olympus BX53 (Japan).

### Isolation of NMs

To obtain NMs by the homogenization method, frozen neutrophil suspensions were thawed and washed with PBS three times (centrifugation at 800 g). Neutrophils were then suspended in hypotonic lysing buffer containing 225 mM d-mannitol, 30 mM Tris-HCl (pH 7.5), 75 mM sucrose, 0.2 mM EGTA, and a protease inhibitor cocktail. Neutrophils were then disrupted using a Dounce homogenizer with a tight-fitting pestle (40 passes). The homogenized solution was centrifuged at 20,000 g for 25 min at 4 °C. The pellet was discarded, and the supernatant was centrifuged again at 120,000 g and 4 ºC for 60 min. To obtain NMs by hypotonic treatment, neutrophils were first treated with hypotonic lysis buffer on ice for 30 min. The supernatants were pooled and centrifuged at 20,000 g and 4 °C for 20 min. Afterward, the pellet was discarded, and the supernatant was centrifuged again at 120,000 g for 60 min at 4 °C. To obtain NMs by the repeated freeze‒thaw method, 1 mL of precooled double-distilled water was added to resuspend the neutrophils. The mixture was then sonicated at a frequency of 42 kHz for 10 min and subjected to 3 freeze‒thaw cycles. It was first frozen at − 80 °C for 30 min and then thawed at room temperature for another 30 min. After centrifugation at 10,000 g for 10 min at 4 °C, the supernatant was collected and then centrifuged at 120,000 g for another 45 min. Following the centrifugation, NMs isolated by three different methods were collected as the pellet and stored at − 80 ºC for further use. NM protein content was quantified using a Lowry Protein Concentration Determination Kit with a bovine serum albumin standard.

### Synthesis and characterization of NM@F127 and R4F-NM@F127

Pluronic F127 polymer loaded with hydrophobic drugs was prepared by thin-film hydration, further mixed with NMs and extruded 20 times through 400 nm and 100 nm polycarbonate membranes with a liposome extruder to prepare NM@F127 nanoparticles. Subsequently, NM@F127 was functionalized with R4F peptide that was capable of binding phospholipids on NMs to prepare R4F-NM@F127. The mean particle size distribution, zeta potential, and PDI of NM@F127 and R4F-NM@F127 were determined with a Malvern Particle Size Analyzer (Malvern Instruments Ltd., Nano-ZS90, Malvern, Worcestershire, UK). The morphology of R4F-NM@F127 was examined using transmission electron microscopy (TEM, JEOL F200, Japan). The absorption wavelength of DiR-BOA-loaded R4F-NM@F127 was measured with a UV‒visible spectrophotometer (AOE Instruments, China).

### High-performance liquid chromatography (HPLC)

To calculate the encapsulation efficiency of NM@F127-Cel and R4F-NM@F127-Cel, HPLC was used to investigate the linear relationship between the Cel concentration and the peak area. The chromatographic conditions for celastrol measurement were as follows: mobile phase: methanol/water = 87/13 (V/V), sample size: 40 µL, flow rate: 1.0 mL/min, separation column temperature: 35 ℃, and detection wavelength: 425 nm.

### Isolation of BMDMs

The mice were sacrificed and rinsed in 75% alcohol for 5 min. The tibia and femur of the mice were removed in a sterile environment, and the epiphysis at both ends of the bones were cut to facilitate needle insertion. The bone marrow cavity was repeatedly flushed with serum-free DMEM until the bone cavity became white. The bone marrow cells were centrifuged at 1350 rpm for 10 min at room temperature. After discarding the supernatant, ACK lysis buffer was added and incubated for 3 min. Then, DMEM was added to stop the reaction, and the cells were centrifuged and resuspended in the appropriate medium. Filter the suspension using a sterile 70 μm cell strainer. After centrifugation, the cells were cultured in DMEM containing 10% FBS, 100 U/mL penicillin‒streptomycin, 50 µM β-mercaptoethanol and 20 ng/mL murine macrophage colony-stimulating factor.

### In vitro targeting test

To assess the SR-B1 receptor-targeting ability of R4F-NM@F127, cells were seeded into 96-well plates at a density of 1.5 × 10^4^ cells/mL and cultured overnight at 37 °C in a humidified 5% CO_2_ atmosphere. After removing the medium, DiR-BOA-labeled NM@F127 and R4F-NM@F127 were added at final concentrations of 2.5 µM, 5 µM, 10 µM and 20 µM and incubated for 3 h. After incubation, the cells were digested with trypsin and washed three times with PBS, and the fluorescence signal intensity of the cells was measured by FCM. For immunofluorescence analysis, cells (3 × 10^4^) were seeded into 8-well chambers to cover the glass bottoms. Then, the cells were incubated with NM@F127 and R4F-NM@F127 at a DiR-BOA concentration of 20 µM for 3 h. The cells were rinsed gently with sterile PBS three times and fixed with 4% paraformaldehyde for 15 min on ice. Cell culture medium containing 5 µg/mL phalloidin was added, and the cells were stained at room temperature for 30 min. Then, the cells were washed three times with PBS. Subsequently, the nuclei were stained with 0.5 µg/mL DAPI for 10 min and rinsed gently with PBS 3 times. Fluorescence images were acquired using a confocal laser scanning microscope with an excitation wavelength of 405 nm for DAPI, 488 nm for FITC and 633 nm for DiR-BOA.

### Cell treatment

RAW264.7 cells were seeded into 6-well plates at a density of 1 × 10^6^ cells/mL and divided into the control, LPS, free Cel, NM@F127-Cel and R4F-NM@F127-Cel groups. After overnight incubation, the cells were incubated with NM@F127-Cel and R4F-NM@F127-Cel for 30 min, followed by stimulation with LPS (100 ng/mL); 2 mL of medium was added at a concentration of 0.25 µM or 0.5 µM, and the normal group and the LPS group were given the same amount of culture medium for 6 h to analyze the mRNA expression of the M1 macrophage polarization markers iNOS, TNF-α, IL-6 and the M2 macrophage polarization markers IL-10 and Arg-1. Similarly, the cells were also treated with Cel, NM@F127-Cel or R4F-NM@F127-Cel for 12 h to analyze M1/M2 macrophage polarization markers and proteins related to the NF-κB and MAPK signaling pathways. The nuclear and cytoplasmic proteins of RAW264.7 cells were extracted using a Nuclear and Cytoplasmic Protein Extraction Kit. The expression levels of p65 in the nuclear and cytoplasmic of RAW264.7 cells were determined by western blotting.

### ELISA assay

The cell supernatants of the above groups were collected, and then the levels of cytokines (TNF-α, IL-6, and IL-10) in the cell supernatants of each group were detected with an ELISA kit according to the manufacturer’s instructions. In addition, blood samples were collected from mice on the day of sacrifice. After incubation at room temperature for 2 h, whole blood was centrifuged at 3000 rpm for 10 min. Serum levels of the inflammatory cytokines TNF-α, IL-6 and IL-10 were determined using ELISA kits.

### Western blot analysis

Proteins extracted from cell lysates were separated by 10% SDS‒PAGE and then transferred to polyvinylidene difluoride (PVDF) membranes. After blocking with 5% skim milk powder for 1.5 h, the proteins were hybridized with the primary antibodies against β-actin (1:1000), iNOS (1:1000), Arg-1 (1:5000), Lamin B (1:500), p65 (1:5000), p-p65 (1:500), IκBα (1:500), p-IκBα (1:500), JNK (1:6000), p-JNK (1:2000), ERK (1:1000) and p-ERK (1:5000) overnight at 4 °C. The membrane was washed three times with TBST buffer and incubated with a goat anti-rabbit IgG H&L (HRP) secondary antibody (1:5000) for 1 h at room temperature. After washing with TBST, proteins detection was performed using ECL reagent and a ChemiScope 6100 chemiluminescence imaging system (Clinx, Shanghai, China). Quantitative analysis of bands was performed at least three times. In western blotting experiments, Lamin B was used as a control for nuclear proteins, and β-actin was used as a control for other proteins.

### Real-time qPCR

Total RNA was extracted from cells using TRIzol reagent and reverse transcribed into cDNA using a reverse transcription kit. Real-time quantitative polymerase chain reaction (RT‒qPCR) was performed on the StepOnePlus RT‒qPCR System using SYBR green. The primer sequences utilized for amplification are shown in Additional file 1: Table S1. Relative expression was calculated by using the 2 ^−△△Ct^ method with normalization to β-actin values. Reactions were repeated a minimum of three times in triplicate. Additional file 1: Table S1. shows the primer sequences used for amplification.

### Flow cytometry analysis

To assess M1/M2 polarization, RAW264.7 cells were seeded in 96-well plates at 1.5 × 10^4^ cells per well and incubated overnight in complete culture medium. LPS (100 ng/mL) was added for 30 min of incubation, except for the normal group. Then, the cells were treated with fresh complete culture medium containing Cel or NM@F127-Cel or R4F-NM@F127-Cel at a dose of 0.25 µM or 0.5 µM for 24 h, respectively. After incubation, RAW264.7 cells were incubated with CD16/32 for 10 min to block Fc receptors, followed by staining with an APC anti-mouse CD86 antibody and a PerCP/Cyanine5.5 anti-mouse CD206 antibody for 30 min and detection using FCM. For macrophages in the synovial fluid of the joint, first, a syringe was used to inject sterile PBS into the joint cavity, and then the liquid inside was extracted from the mice. The cells were filtered through a 70 μm cell strainer and then washed once with PBS to prepare single-cell suspensions. The single-cell suspensions were incubated with CD16/32 for 10 min to block Fc receptors, followed by staining with an APC/Cyanine7 anti-mouse F4/80 antibody, and a PE/Cyanine7 anti-mouse/human CD11b antibody, APC anti-mouse CD86 antibody, PerCP/Cyanine5.5 anti-mouse CD206 antibody for 30 min and detection using a flow cytometer (Dakewe Biotech Co., Ltd., China). The data were analyzed using FlowJo software (FlowJo, Ashland, OR, USA).

### Immunofluorescence staining

For immunofluorescence staining, RAW264.7 cells were fixed with 4% paraformaldehyde for 20 min at room temperature and permeabilized with 0.3% Triton X-100 for 10 min. Then, the cells were washed three times with PBS. Subsequently, the cells were blocked for 30 min at room temperature with 1% bovine serum albumin containing 0.1% Triton X-100. After that, the cells were incubated overnight with an anti-NF-kB p65 antibody (1:100). Following incubation, the cells were rinsed three times with PBS and incubated with a goat anti-rabbit IgG H&L (Alexa Fluor® 594) (1:400) antibody in the dark for 1 h at room temperature. The nuclei were stained with DAPI. Fluorescence images were acquired using a confocal laser scanning microscope (A1R, Nikon, Japan). The data were analyzed using ImageJ software. For immunofluorescence staining of the synovium of the knee joint, freshly harvested knee joints were fixed with 4% paraformaldehyde for 24 h and then decalcified with PBS containing 15% EDTA at 4 ℃ for three days, and the decalcification solution was changed daily. Once decalcification was complete, the samples were washed overnight at 4 ℃. Then, the tissues were cryoprotected in 30% sucrose in PBS at 4 ℃ until they sank and embedded in OCT compound before freezing on dry ice. Tissue Sect. (10 μm thick) were cut on a cryostat (Dakewe Biotech Co., Ltd., China) and mounted on poly-L-lysine-coated slides. After blocking with 1% bovine serum albumin for 1 h, an iNOS polyclonal antibody (1:100) or Arginase-1 polyclonal antibody (1:100) was added and incubated overnight at 4 ℃. Following incubation, the tissue sections were rinsed three times with PBS and incubated with a goat anti-rabbit IgG H&L (Alexa Fluor® 594) (1:400) antibody in the dark for 1 h at room temperature. The nuclei were stained with DAPI. All sections were imaged using a confocal laser scanning microscope with a dry 20×/0.8 NA objective. The data were analyzed using ImageJ software.

### Establishing a mouse model of CIA

The collagen-induced arthritis (CIA) animal model was established in DBA/1 mice per the manufacturer’s instructions (Chondrex, USA). Briefly, bovine type II collagen (2 mg/mL) was thoroughly emulsified with an equal volume of complete Freund’s adjuvant (2 mg/mL) by using a T-branch pipe, and 100 µL of the emulsion was administered to mice intradermally at the base of the tail. On day 21 after primary injection, the mice received an intradermal booster injection of type II collagen with an equal volume of incomplete Freund’s adjuvant.

### In vivo fluorescence targeting test

A total of 12 CIA mice were randomly divided into 4 groups (3 animals per group), which were intravenously administered DiR-BOA-labeled F127, NM@F127, R4F-NM@F127, or free DiR-BOA (20 nmol). After 1, 3, 6, 12 and 24 h, the mice were anesthetized and imaged with a small animal in vivo imaging system (IVIS Lumina XRMS). After 24 h, the mice were euthanized, and the blood, heart, liver, spleen, lung, kidney, brain and joint were removed. Blood was sampled and centrifuged at 3000 g for 10 min to obtain plasma. Synovial fluid was collected in a tube. The fluorescence of plasma, synovial fluid, and organs was measured using a small animal in vivo imaging system (IVIS Lumina XRMS). To further evaluate the colocalization of nanoparticles with synovial fluid macrophages, the mean fluorescence intensity of DiR-BOA in synovial fluid macrophages was detected by FCM. Confocal microscopy verified that DiR-BOA-labeled R4F-NM@F127 colocalized with synovial macrophages.

### Therapeutic effects in CIA mice

Twenty-eight days after the first immunization, mice with CIA were randomly divided into four groups (n = 4): the CIA model group, free Cel-treated group, NM@F127-Cel-treated group and R4F-NM@F127-Cel-treated group. The three treatment groups were then administered via the tail vein at a dose of 5 mg/kg body weight of Cel on days 28 and 38. The model group was injected with an equal volume of PBS by the same method and on the same days. Normal DBA/1 mice were maintained as the control group. Mice were weighed, and the paw thickness of both hind ankle joints was measured with a Vernier caliper and examined for clinical scores every 3 days from day 21. The position of the caliper was the same during each measurement. Paw inflammation was scored visually as follows: 0 = normal paw; 1 = one toe inflamed and swollen; 2 = more than one toe but not the entire paw inflamed and swollen, or mild swelling of entire paw; 3 = erythema and moderate swelling extending to the entire paw; and 4 = severe erythema and swelling of the whole paw and ankle. Each paw was graded with a score from 0 to 4, generating an arthritis score on a scale of 0–16 for each individual mouse. Next, to evaluate joint swelling, the hind paws of the mice were photographed on day 18 (3 days before secondary immunization), day 28 (on treatment day), day 35 (7 days after treatment), day 42 (14 days after treatment) and day 45 with a camera.

### Micro-computed tomography (Micro-CT) analyses

Scanning of mouse paws was performed on a Nemo Micro-CT (NMC-200) device from PINGSENG Healthcare Inc. The device uses cone beam CT (cone beam CT) technology, which is an imaging technology that can achieve high resolution. At the beginning of the experiment, the mouse paw was placed vertically into the sample chamber, the scanning tube voltage was set to 60 kV, and the tube current was set to 120 µA. During the scanning process, the detector and the bulb rotated 360° around the central axis of the sample chamber, and in the scanning area, 4000 projections were carried out within the 1200 s scan time. After the image was captured by the detector, it was transferred to a computer, and the image was reverse reconstructed using the FDK method in Avatar software with a pixel size of 8 μm × 8 μm × 9 μm.

### Histopathologic analysis of the joints

After the CIA mice were sacrificed on day 45, the hind legs were removed. The knee and ankle joints were fixed and decalcified. Then, the decalcified limbs were dehydrated step by step and embedded in paraffin. Paraffin sections of murine paws were stained with hematoxylin and eosin (H&E) and safranin O (S&O) to evaluate of inflammation and joint destruction. All images were captured and analyzed with an Olympus BX53 microscope (Japan).

### Toxicological evaluation

The serum toxicological indexes aspartate aminotransferase (AST) and alanine aminotransferase (ALT) were assayed using a commercial kit according to the manufacturer’s protocol. For histopathological observation, samples from the heart, liver, spleen, lung, kidney and brain were harvested. The tissues were fixed in 4% paraformaldehyde for paraffin sectioning followed by H&E staining, and all images were captured and analyzed with an Olympus BX53 microscope (Japan).

### Statistical analysis

Statistical analysis was performed using GraphPad Prism (GraphPad Software 8.0.2). Data are presented as the mean ± SEM. If the data conformed to a normal distribution and homogeneity of variance, the experimental results between the two groups were compared by independent sample t test, and the experimental results between multiple groups were compared by one-way analysis of variance (one-way ANOVA). Significant differences between or among the groups are indicated as follows: ns represents no significant difference, *p < 0.05, **p < 0.01, ***p < 0.001; ^#^p < 0.05, ^##^p < 0.01 and ^###^p < 0.001.

## Supplementary information

**Additional file 1: Fig. S1 a** Schematic diagram of neutrophil isolation from the peripheral blood of mice. **b** Neutrophils were identified by using Giemsa staining. **Fig. S2 a-c** Comparison of protein concentration (**a**), cell membrane vesicle number (**b**) and average diameter (**c**) for three methods for separating neutrophil membrane vesicles. **d** The adhesion molecule LFA-1 on the neutrophils and neutrophil membrane vesicles were detected by western blot. **Fig. S3** The polydispersity index (PDI) of NM@f127 and R4F-NM@F127. **Fig. S4** Confocal imaging of the ability of DiR-BOA-labeled R4F-NM@F127 to target RAW264.7 cells at 37 °C or 4 °C in vitro. **Fig. S5** Quantitative analysis of the radiant efficiency in organs. **Fig. S6 a** Ex vivo imaging of the serum at 24 h after intravenous injection. **b** Quantitative analysis of the radiant efficiency with DiR-BOA-loaded R4F-NM@F127, NM@F127, F127 and free DiR-BOA in serum. **Fig. S7 a** Average size of NM@F127-Cel and R4F-NM@F127-Cel. **b** Zeta potential of NM@F127-Cel and R4F-NM@F127-Cel. **Fig. S8** White light image and UV-Vis absorption spectrum of R4F-NM@F127, free Cel and R4F-NM@F127-Cel. **Fig. S9** RT-qPCR analysis of mRNA expression of M1 and M2 macrophage markers in LPS-induced RAW264.7 cells after different treatments. **Fig. S10** Quantitative analysis of iNOS and Arg-1 protein levels in LPS-induced RAW264.7 cells after different treatments. **Fig. S11** Quantitative analysis the protein levels of p65 in the nucleus and cytoplasm in LPS-induced RAW264.7 cells after different treatments. **Fig. S12** The dates of clinical scores (**a**), and ankle diameter (mm) of the left hind paw (**b**) and the right hind paw (**c**) of mice were counted on days 45. **Fig. S13** Representative ankle histopathology pictures of H&E staining and S&O staining. **Fig. S14** H&E staining of heart, liver, spleen, lung, kidney and brain extracted at 45^st^ day after the first immunization. **Fig. S15** Levels of ALT (**a**) and AST (**b**) in serum. **Table S1.** Primer sequences for the amplification.

## Data Availability

The data that support the findings of this study are available from the corresponding author upon reasonable request.

## References

[1] Chen Z, Bozec A, Ramming A, Schett G (2019). Anti-inflammatory and immune-regulatory cytokines in rheumatoid arthritis. Nat Rev Rheumatol.

[2] Alam J, Jantan I, Bukhari SNA (2017). Rheumatoid arthritis: recent advances on its etiology, role of cytokines and pharmacotherapy. Biomed Pharmacother.

[3] Di Benedetto P, Ruscitti P, Vadasz Z, Toubi E, Giacomelli R (2019). Macrophages with regulatory functions, a possible new therapeutic perspective in autoimmune diseases. Autoimmun Rev.

[4] Siouti E, Andreakos E (2019). The many facets of macrophages in rheumatoid arthritis. Biochem Pharmacol.

[5] Udalova IA, Mantovani A, Feldmann M (2016). Macrophage heterogeneity in the context of rheumatoid arthritis. Nat Rev Rheumatol.

[6] Wang Y, Chen S, Du K, Liang C, Wang S, Owusu Boadi E, Li J, Pang X, He J, Chang YX (2021). Traditional herbal medicine: therapeutic potential in rheumatoid arthritis. J Ethnopharmacol.

[7] Liu X, Wang Z, Qian H, Tao W, Zhang Y, Hu C, Mao W, Guo Q (2022). Natural medicines of targeted rheumatoid arthritis and its action mechanism. Front Immunol.

[8] An L, Li Z, Shi L, Wang L, Wang Y, Jin L, Shuai X, Li J (2020). Inflammation-targeted celastrol nanodrug attenuates collagen-Induced arthritis through NF-kappaB and notch1 pathways. Nano Lett.

[9] Kim H, Back JH, Han G, Lee SJ, Park YE, Gu MB, Yang Y, Lee JE, Kim SH (2022). Extracellular vesicle-guided in situ reprogramming of synovial macrophages for the treatment of rheumatoid arthritis. Biomaterials.

[10] Yang Y, Guo L, Wang Z, Liu P, Liu X, Ding J, Zhou W (2021). Targeted silver nanoparticles for rheumatoid arthritis therapy via macrophage apoptosis and re-polarization. Biomaterials.

[11] Yan F, Zhong Z, Wang Y, Feng Y, Mei Z, Li H, Chen X, Cai L, Li C (2020). Exosome-based biomimetic nanoparticles targeted to inflamed joints for enhanced treatment of rheumatoid arthritis. J Nanobiotechnol.

[12] Gong T, Tan T, Zhang P, Li H, Deng C, Huang Y, Gong T, Zhang Z (2020). Palmitic acid-modified bovine serum albumin nanoparticles target scavenger receptor-A on activated macrophages to treat rheumatoid arthritis. Biomaterials.

[13] Jones IA, Togashi R, Wilson ML, Heckmann N, Vangsness CT (2019). Intra-articular treatment options for knee osteoarthritis. Nat Rev Rheumatol.

[14] Wang Q, Sun X (2017). Recent advances in nanomedicines for the treatment of rheumatoid arthritis. Biomater Sci.

[15] Wright HL, Moots RJ, Edwards SW (2014). The multifactorial role of neutrophils in rheumatoid arthritis. Nat Rev Rheumatol.

[16] Jeong J, Kim YJ, Lee DY, Moon BG, Sohn KY, Yoon SY, Kim JW (2019). 1-Palmitoyl-2-linoleoyl-3-acetyl-rac-glycerol (PLAG) attenuates gemcitabine-induced neutrophil extravasation. Cell Biosci.

[17] Zhu LM, Zeng D, Lei XC, Huang J, Deng YF, Ji YB, Liu J, Dai FF, Li YZ, Shi DD (2020). KLF2 regulates neutrophil migration by modulating CXCR1 and CXCR2 in asthma. Biochim Biophys Acta Mol Basis Dis.

[18] Teijeira A, Garasa S, Ochoa MDC, Cirella A, Olivera I, Glez-Vaz J, Andueza MP, Migueliz I, Alvarez M, Rodriguez-Ruiz ME (2021). Differential Interleukin-8 thresholds for chemotaxis and netosis in human neutrophils. Eur J Immunol.

[19] Han Y, Zhao R, Xu F (2018). Neutrophil-based delivery systems for nanotherapeutics. Small.

[20] Bi YH, Duan WX, Chen J, You T, Li SY, Jiang W, Li M, Wang G, Pan XY, Wu J (2021). Neutrophil decoys with anti-inflammatory and anti-oxidative properties reduce secondary Spinal cord injury and improve neurological functional recovery. Adv Funct Mater.

[21] Wang D, Wang SY, Zhou ZD, Bai D, Zhang QZ, Ai XZ, Gao WW, Zhang LF (2022). White blood cell membrane-coated nanoparticles: recent development and medical applications. Adv Healthcare Mater.

[22] Lu LS, Qi SH, Chen YZ, Luo HM, Huang SL, Yu X, Luo QM, Zhang ZH (2020). Targeted immunomodulation of inflammatory monocytes across the blood-brain barrier by curcumin-loaded nanoparticles delays the progression of experimental autoimmune encephalomyelitis. Biomaterials.

[23] Lin J, He Y, Wang B, Xun Z, Chen S, Zeng Z, Ou Q (2019). Blocking of YY1 reduce neutrophil infiltration by inhibiting IL-8 production via the PI3K-Akt-mTOR signaling pathway in rheumatoid arthritis. Clin Exp Immunol.

[24] Kurowska-Stolarska M, Alivernini S (2022). Synovial tissue macrophages in joint homeostasis, rheumatoid arthritis and disease remission. Nat Rev Rheumatol.

[25] Headland SE, Jones HR, Norling LV, Kim A, Souza PR, Corsiero E, Gil CD, Nerviani A, Dell’Accio F, Pitzalis C (2015). Neutrophil-derived microvesicles enter cartilage and protect the joint in inflammatory arthritis. Sci Transl Med.

[26] Yang X, Chang Y, Wei W (2020). Emerging role of targeting macrophages in rheumatoid arthritis: focus on polarization, metabolism and apoptosis. Cell Prolif.

[27] Liu XS, Guo RR, Huo SC, Chen H, Song QX, Jiang G, Yu Y, Huang JL, Xie SW, Gao XL, Lu LJ (2022). CaP-based anti-inflammatory HIF-1 alpha siRNA-encapsulating nanoparticle for rheumatoid arthritis therapy. J Controlled Release.

[28] Feng K, Chen H, Xu C (2020). Chondro-protective effects of celastrol on osteoarthritis through autophagy activation and NF-kappaB signaling pathway inhibition. Inflamm Res.

[29] Arthur JSC, Ley SC (2013). Mitogen-activated protein kinases in innate immunity. Nat Rev Immunol.

[30] Zhang Y, Gao Z, Chao S, Lu W, Zhang P (2022). Transdermal delivery of inflammatory factors regulated drugs for rheumatoid arthritis. Drug Deliv.

[31] Yu C, Liu H, Guo C, Chen Q, Su Y, Guo H, Hou X, Zhao F, Fan H, Xu H (2022). Dextran sulfate-based MMP-2 enzyme-sensitive SR-A receptor targeting nanomicelles for the treatment of rheumatoid arthritis. Drug Deliv.

