# Supplementary material for: Peptide-anchored neutrophil membrane-coated biomimetic nanodrug for targeted treatment of rheumatoid arthritis
Source: J Nanobiotechnology. 2023 Jan 13;21:13. doi: 10.1186/s12951-023-01773-x (PMC9837964; doi:10.1186/s12951-023-01773-x)
Supplement: Supplementary file 1 — Additional file 1: Fig. S1 a Schematic diagram of neutrophil isolation from the peripheral blood of mice. b Neutrophils were identified by using Giemsa staining. Fig. S2 a-c Comparison of protein concentration (a), cell membrane vesicle number (b) and average diameter (c) for three methods for separating neutrophil membrane vesicles. d The adhesion molecule LFA-1 on the neutrophils and neutrophil membrane vesicles were detected by western blot. Fig. S3 The polydispersity index (PDI) of NM@f127 and R4F-NM@F127. Fig. S4 Confocal imaging of the ability of DiR-BOA-labeled R4F-NM@F127 to target RAW264.7 cells at 37 °C or 4 °C in vitro. Fig. S5 Quantitative analysis of the radiant efficiency in organs. Fig. S6 a Ex vivo imaging of the serum at 24 h after intravenous injection. b Quantitative analysis of the radiant efficiency with DiR-BOA-loaded R4F-NM@F127, NM@F127, F127 and free DiR-BOA in serum. Fig. S7 a Average size of NM@F127-Cel and R4F-NM@F127-Cel. b Zeta potential of NM@F127-Cel and R4F-NM@F127-Cel. Fig. S8 White light image and UV-Vis absorption spectrum of R4F-NM@F127, free Cel and R4F-NM@F127-Cel. Fig. S9 RT-qPCR analysis of mRNA expression of M1 and M2 macrophage markers in LPS-induced RAW264.7 cells after different treatments. Fig. S10 Quantitative analysis of iNOS and Arg-1 protein levels in LPS-induced RAW264.7 cells after different treatments. Fig. S11 Quantitative analysis the protein levels of p65 in the nucleus and cytoplasm in LPS-induced RAW264.7 cells after different treatments. Fig. S12 The dates of clinical scores (a), and ankle diameter (mm) of the left hind paw (b) and the right hind paw (c) of mice were counted on days 45. Fig. S13 Representative ankle histopathology pictures of H&E staining and S&O staining. Fig. S14 H&E staining of heart, liver, spleen, lung, kidney and brain extracted at 45st day after the first immunization. Fig. S15 Levels of ALT (a) and AST (b) in serum. Table S1. Primer sequences for the amplification. [file 12951_2023_1773_MOESM1_ESM.docx]

Peptide-Anchored Neutrophil Membrane-Coated Biomimetic Nanodrug for Targeted Treatment of Rheumatoid Arthritis

Ni Yang^1,2,3†^, Miaomiao Li^1,2,3†^, Ling Wu^4†^, Yinhong Song^2,3†^, Shi Yu^3^, Yingying Wan^1,2,3^, Wenjing Cheng^1,2,3^, Baoye Yang^1,2,3^, Xiaoqin Mou^1,2,3^, Hong Yu^1,2,3^, Jing Zheng^4^, Xinzhi Li^3*^, and Xiang Yu^1,2,3*^

^†^Ni Yang, Miaomiao Li, Ling Wu and Yinhong Song contributed equally to this work.

*Correspondence: yuxiangwl2008@sina.com

^1^ Hubei Key Laboratory of Tumor Microenvironment and Immunotherapy, China Three Gorges University, Yichang 443002, China

^2^ Institute of Infection and Inflammation, China Three Gorges University, Yichang 443002, China

^3^ College of Basic Medical Science, China Three Gorges University, Yichang 443002, China

^4^ The People's Hospital of China Three Gorges University, Yichang 443099, China

**Supplementary Figures**


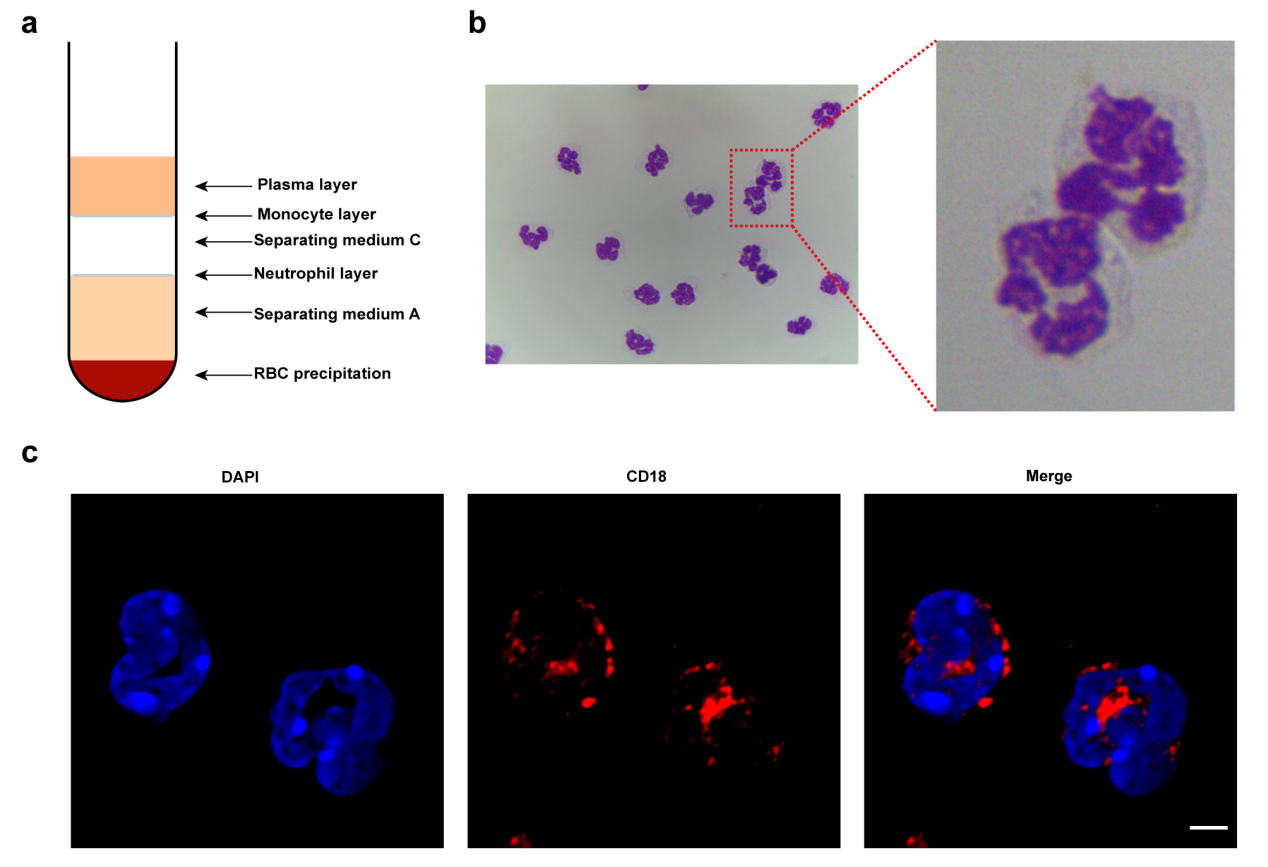


**Fig. S1** **a** Schematic diagram of neutrophil isolation from the peripheral blood of mice. **b** Neutrophils were identified by using Giemsa staining (left, 100×).


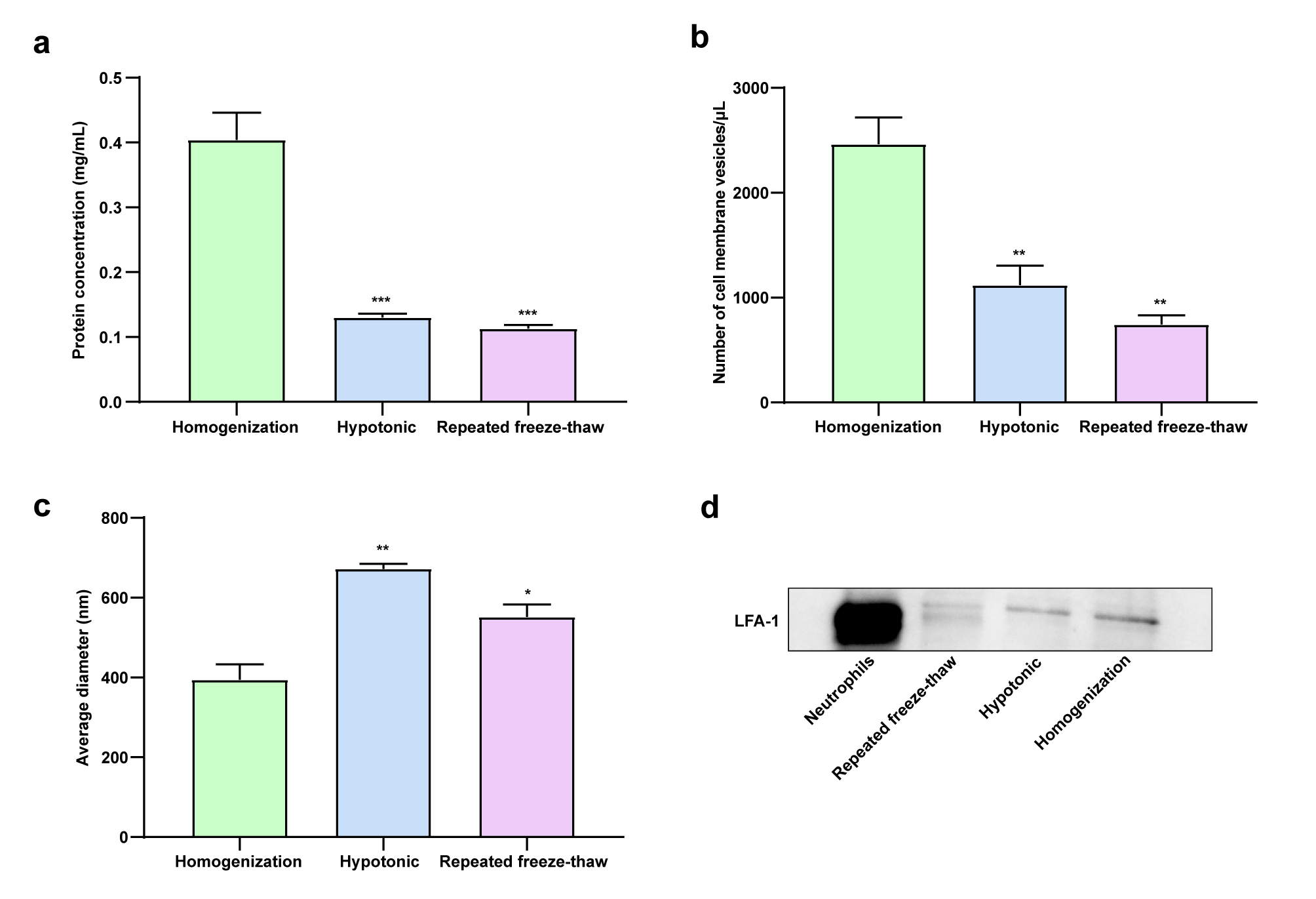


**Fig. S2** **a-c** Comparison of protein concentration (**a**), cell membrane vesicle number (**b**) and average diameter (**c**) for three methods for separating neutrophil membrane vesicles. **d** The adhesion molecule LFA-1 on the neutrophils and neutrophil membrane vesicles were detected by western blot.


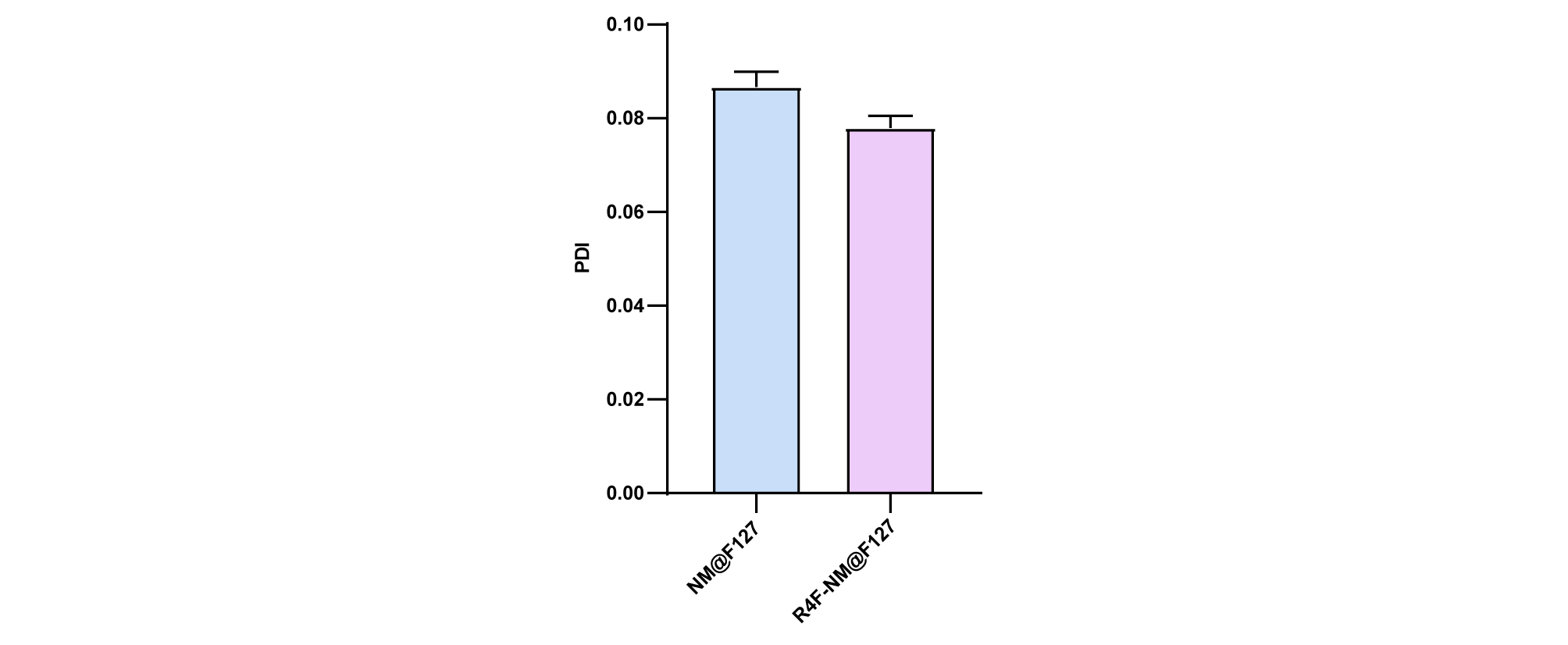


**Fig. S3** The polydispersity index (PDI) of NM@f127 and R4F-NM@F127.


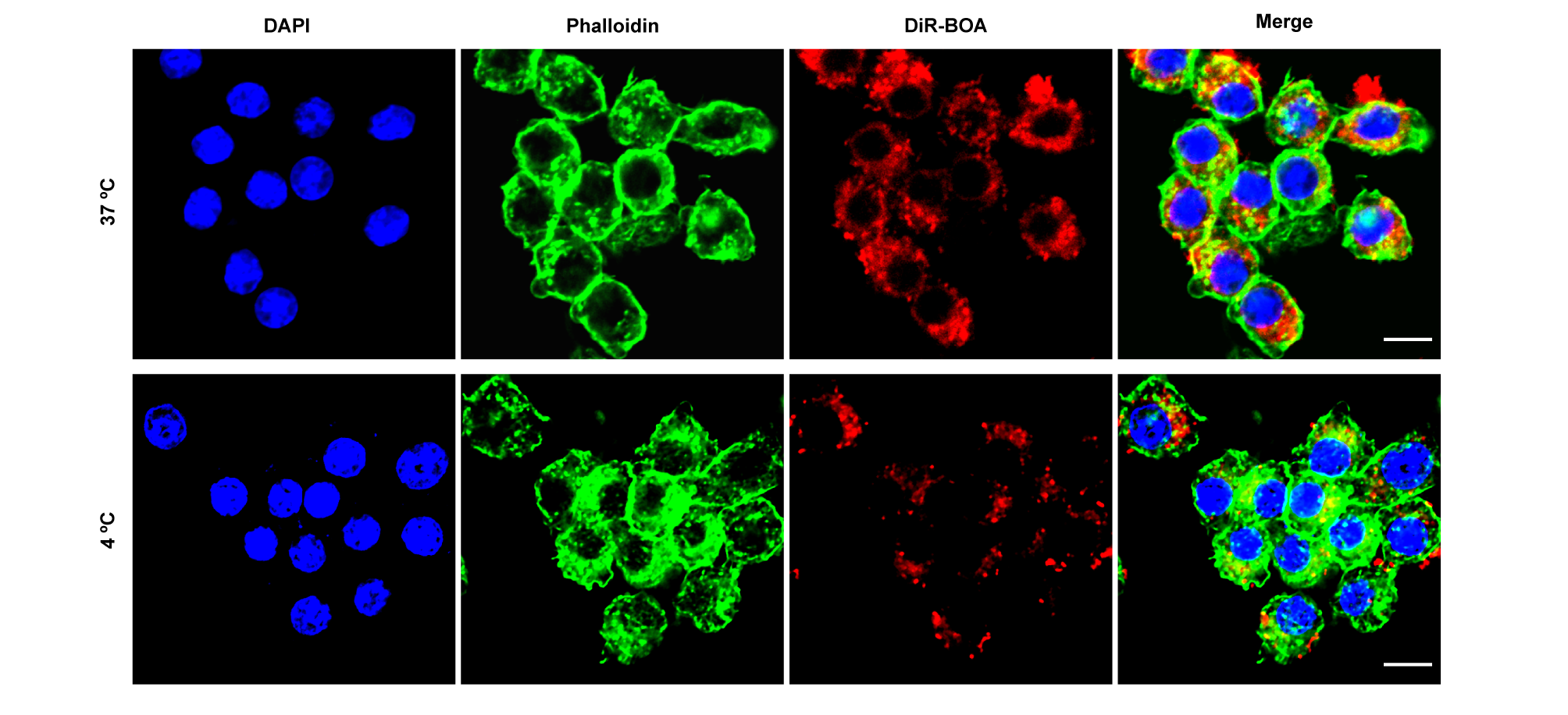


**Fig. S4** Confocal imaging of the ability of DiR-BOA-labeled R4F-NM@F127 to target RAW264.7 cells at 37 °C or 4 °C *in vitro*. Scale bar, 10 μm; Blue, DAPI; Green, phalloidin; Red, DiR-BOA.


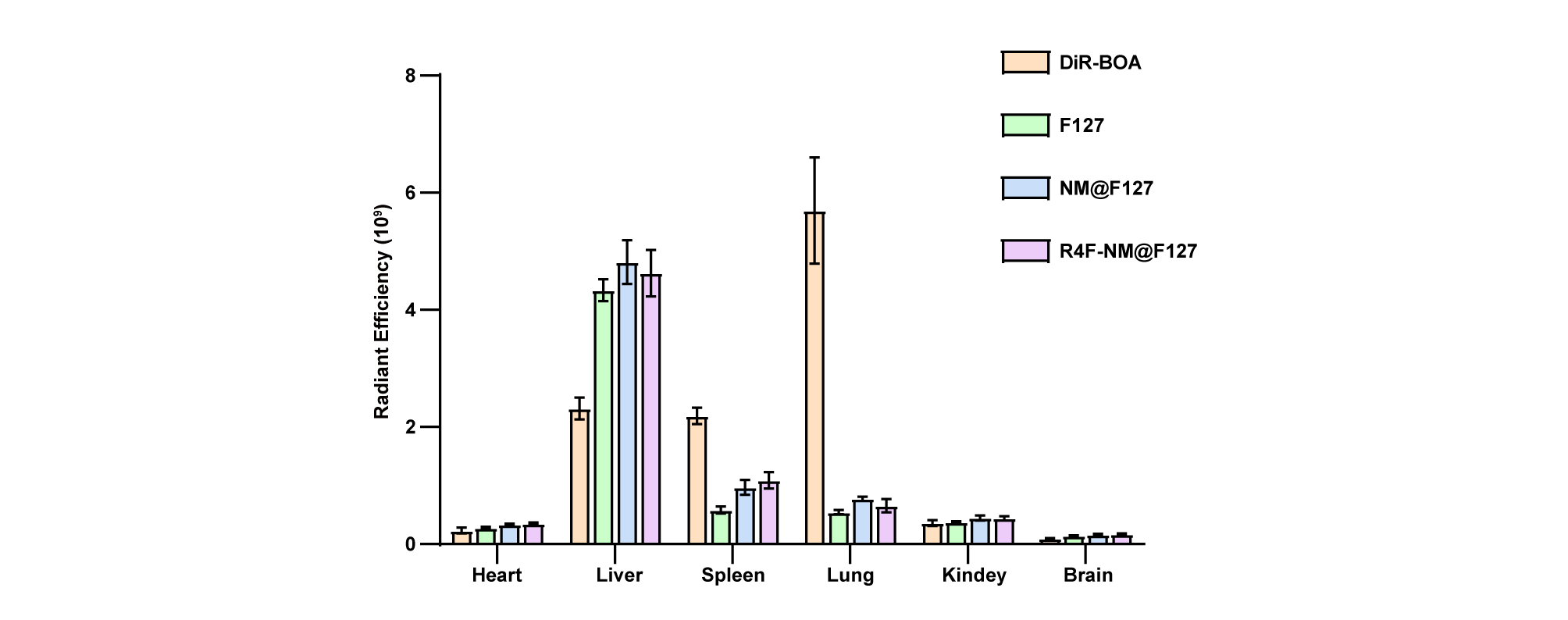


**Fig. S5** Quantitative analysis of the radiant efficiency in organs (n = 3).


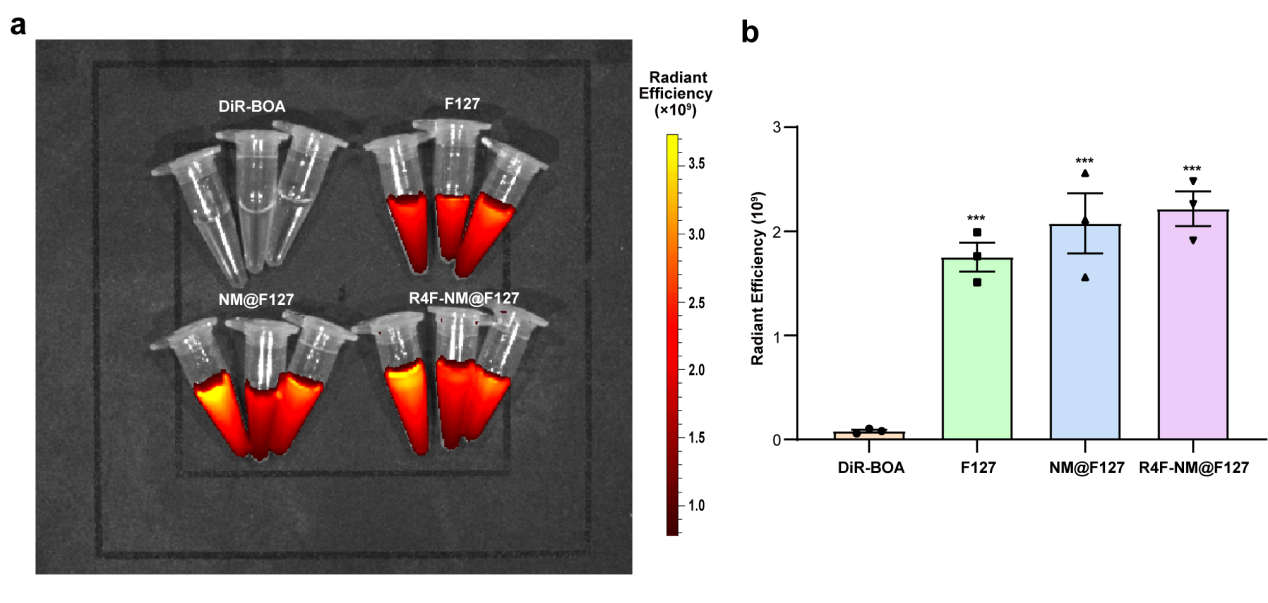


**Fig. S6** **a** Ex vivo imaging of the serum at 24 h after intravenous injection. **b** Quantitative analysis of the radiant efficiency with DiR-BOA-loaded R4F-NM@F127, NM@F127, F127 and free DiR-BOA in serum (n = 3).


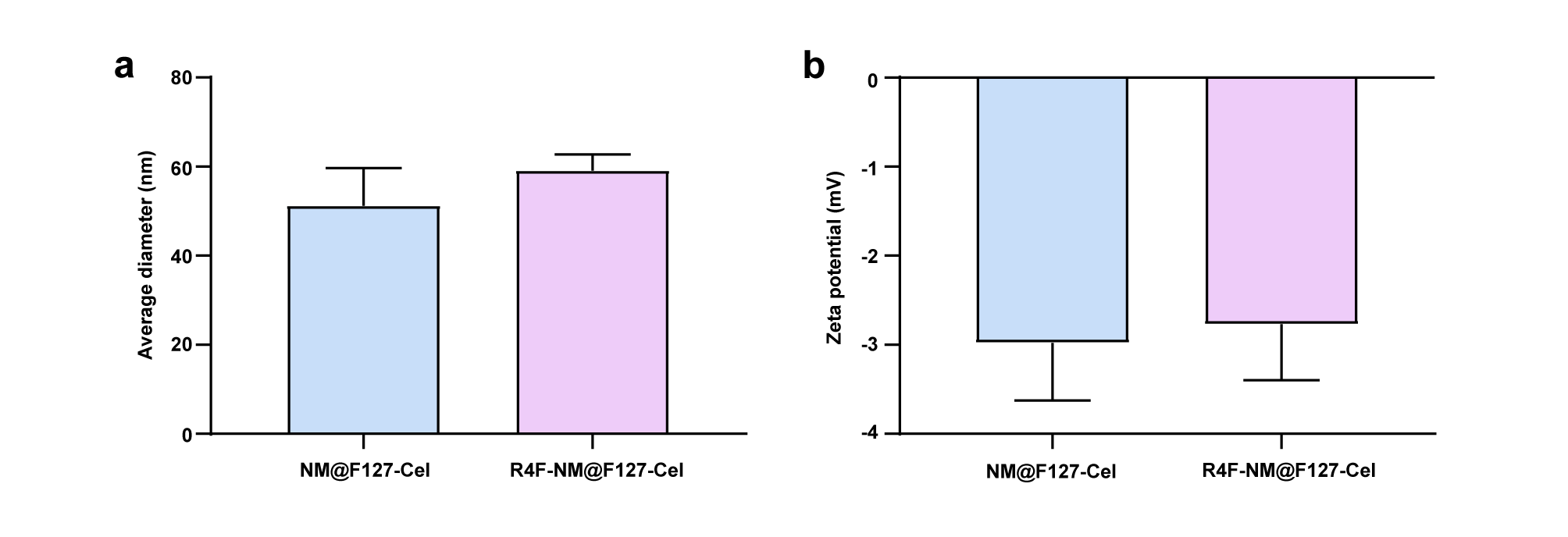


**Fig. S7 a** Average size of NM@F127-Cel and R4F-NM@F127-Cel. **b** Zeta potential of NM@F127-Cel and R4F-NM@F127-Cel. (n = 3).


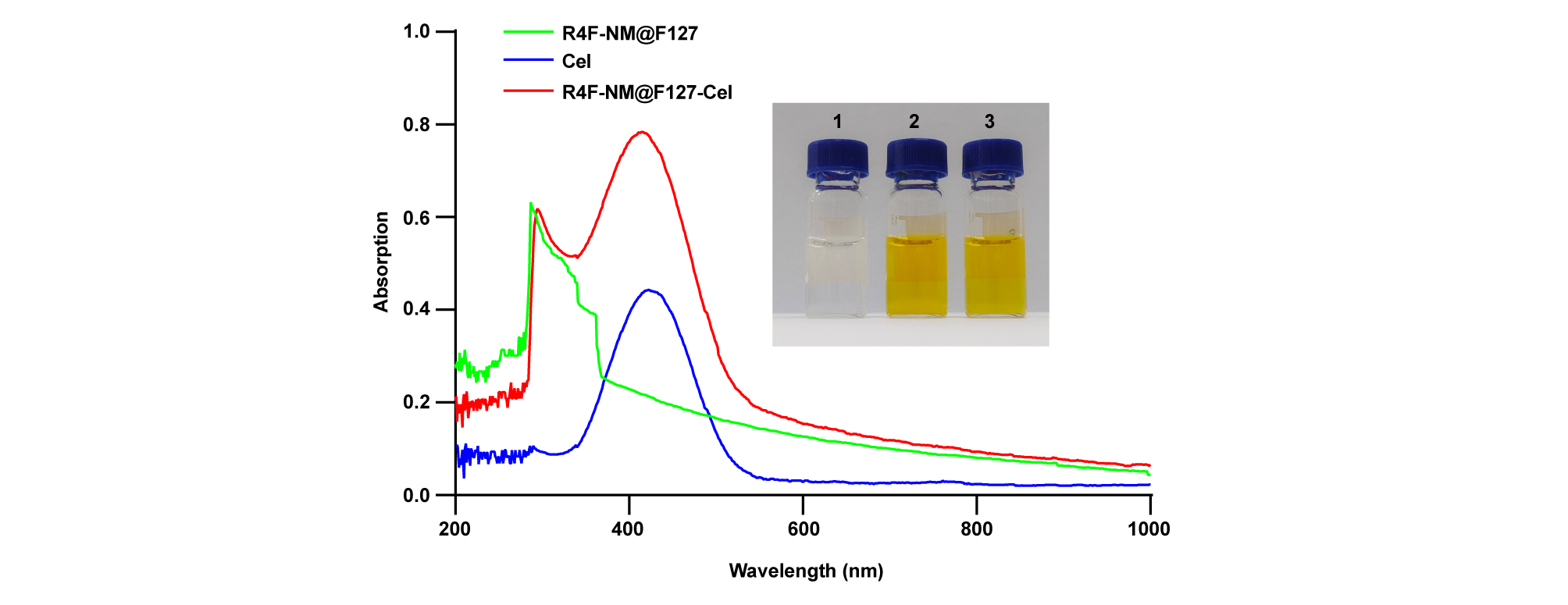


**Fig. S8** White light image and UV-Vis absorption spectrum of R4F-NM@F127, free Cel and R4F-NM@F127-Cel. 1: R4F-NM@F127; 2: free Cel; 3: R4F-NM@F127-Cel.


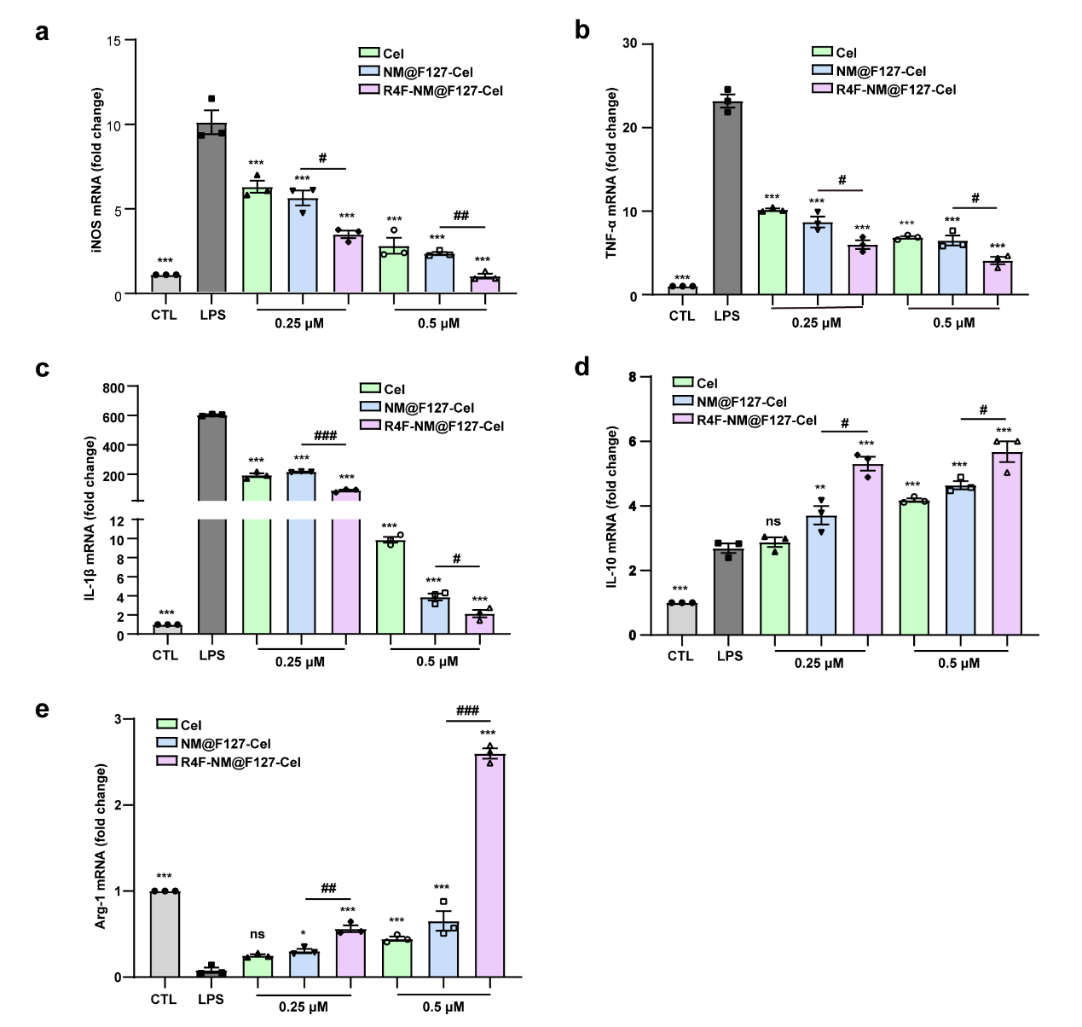


**Fig. S9** **a-c** RT-qPCR analysis of mRNA expression of M1 macrophage markers in LPS-induced RAW264.7 cells after different treatments, including iNOS (**a**), IL-1β (**b**) and TNF-α (**c**). **d-e** RT-qPCR analysis of mRNA expression of M2 macrophage markers, including IL-10 (**d**) and Arg-1 (**e**). Data are presented as the mean ± SEM (n = 3) and were statistically analyzed using one-way ANOVA followed by Tukey’s multiple comparisons test by comparing the group marked * with the LPS group. *p < 0.05, **p < 0.01, ***p < 0.001, ns, not significant; ^#^p < 0.05, ^##^p < 0.01 and ^###^p < 0.001.


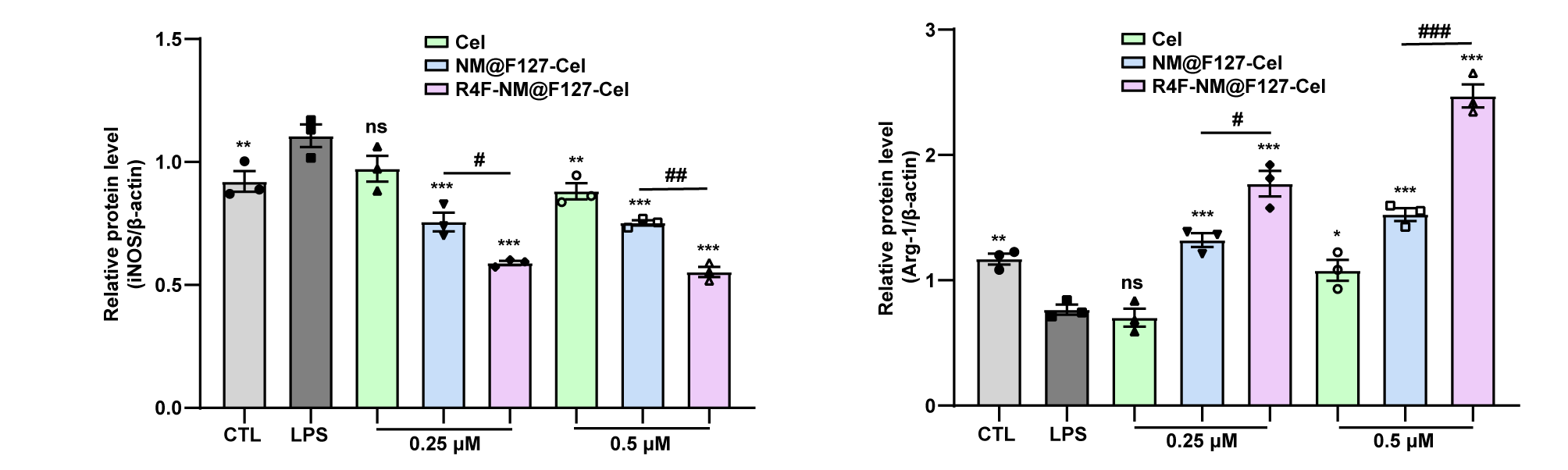


**Fig. S10** Quantitative analysis of iNOS and Arg-1 protein levels in LPS-induced RAW264.7 cells after different treatments. Data are presented as the mean ± SEM (n = 3) and were statistically analyzed using one-way ANOVA followed by Tukey’s multiple comparisons test by comparing the group marked * with the LPS group. *p < 0.05, **p < 0.01, ***p < 0.001. ns represents no significant difference. ^#^p < 0.05, ^##^p < 0.01 and ^###^p < 0.001.


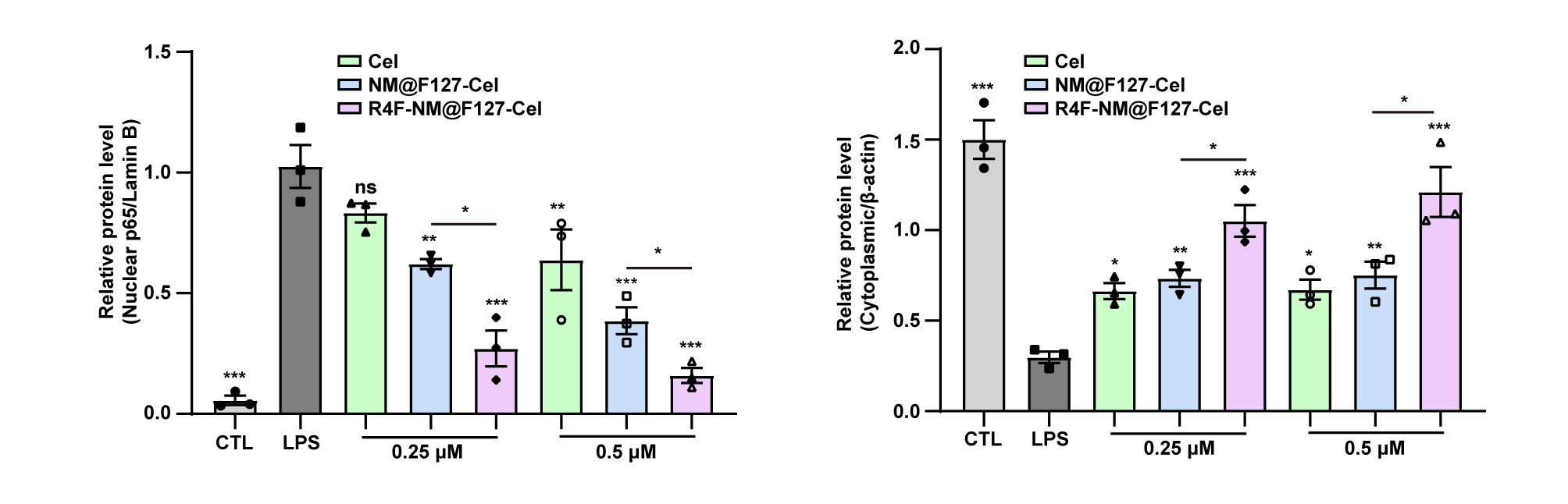


**Fig. S11** Quantitative analysis the protein levels of p65 in the nucleus and cytoplasm in LPS-induced RAW264.7 cells after different treatments. Data are presented as the mean ± SEM (n = 3) and were statistically analyzed using one-way ANOVA followed by Tukey’s multiple comparisons test by comparing the group marked * with the LPS group. *p < 0.05, **p < 0.01, ***p < 0.001. ns represents no significant difference. ^#^p < 0.05, ^##^p < 0.01 and ^###^p < 0.001.

**
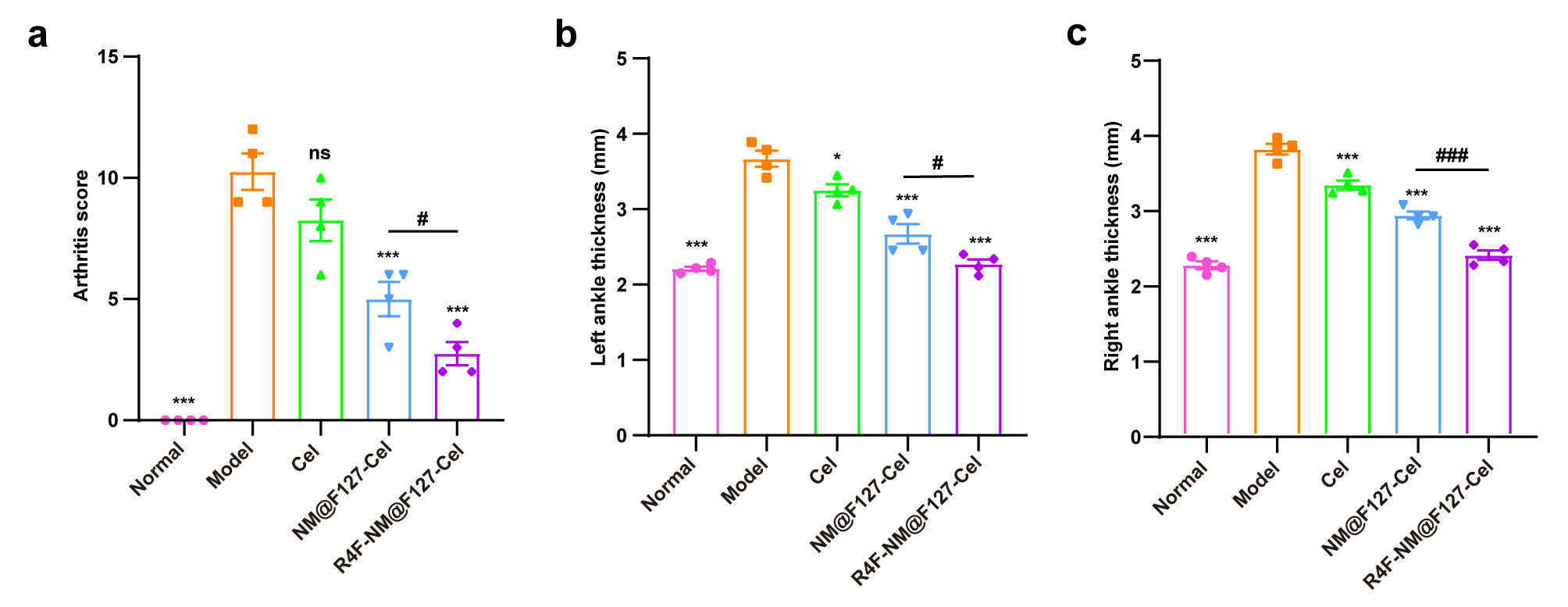
**

**Fig. S12** The dates of clinical scores (**a**), and ankle diameter (mm) of the left hind paw (**b**) and the right hind paw (**c**) of mice were counted on days 45. Data are presented as the mean ± SEM (n = 4) and were statistically analyzed using one-way ANOVA followed by Tukey’s multiple comparisons test by comparing the group marked * with the model group. *p < 0.05, **p < 0.01, ***p < 0.001. ns represents no significant difference. ^#^p < 0.05, ^##^p < 0.01 and ^###^p < 0.001.


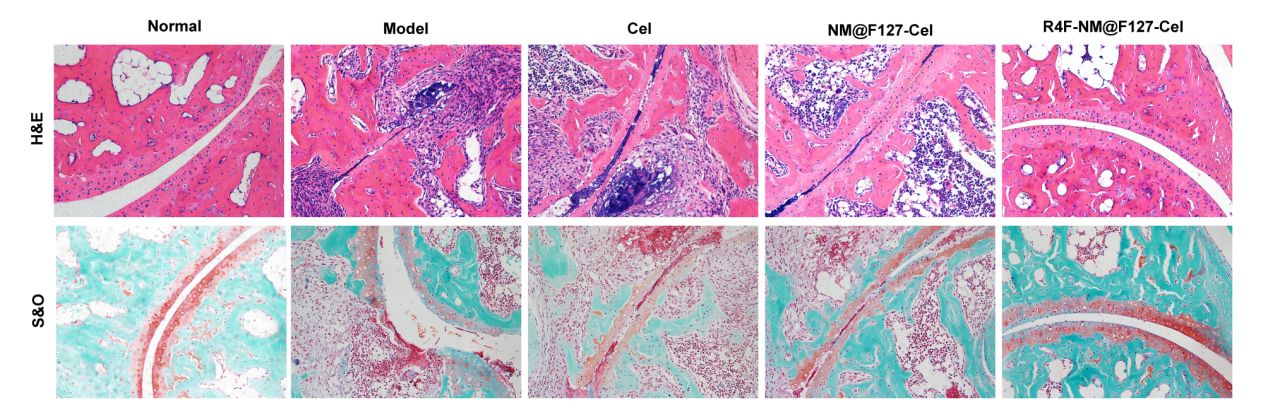


**Fig. S13** Representative ankle histopathology pictures of H&E staining and S&O staining (200×).


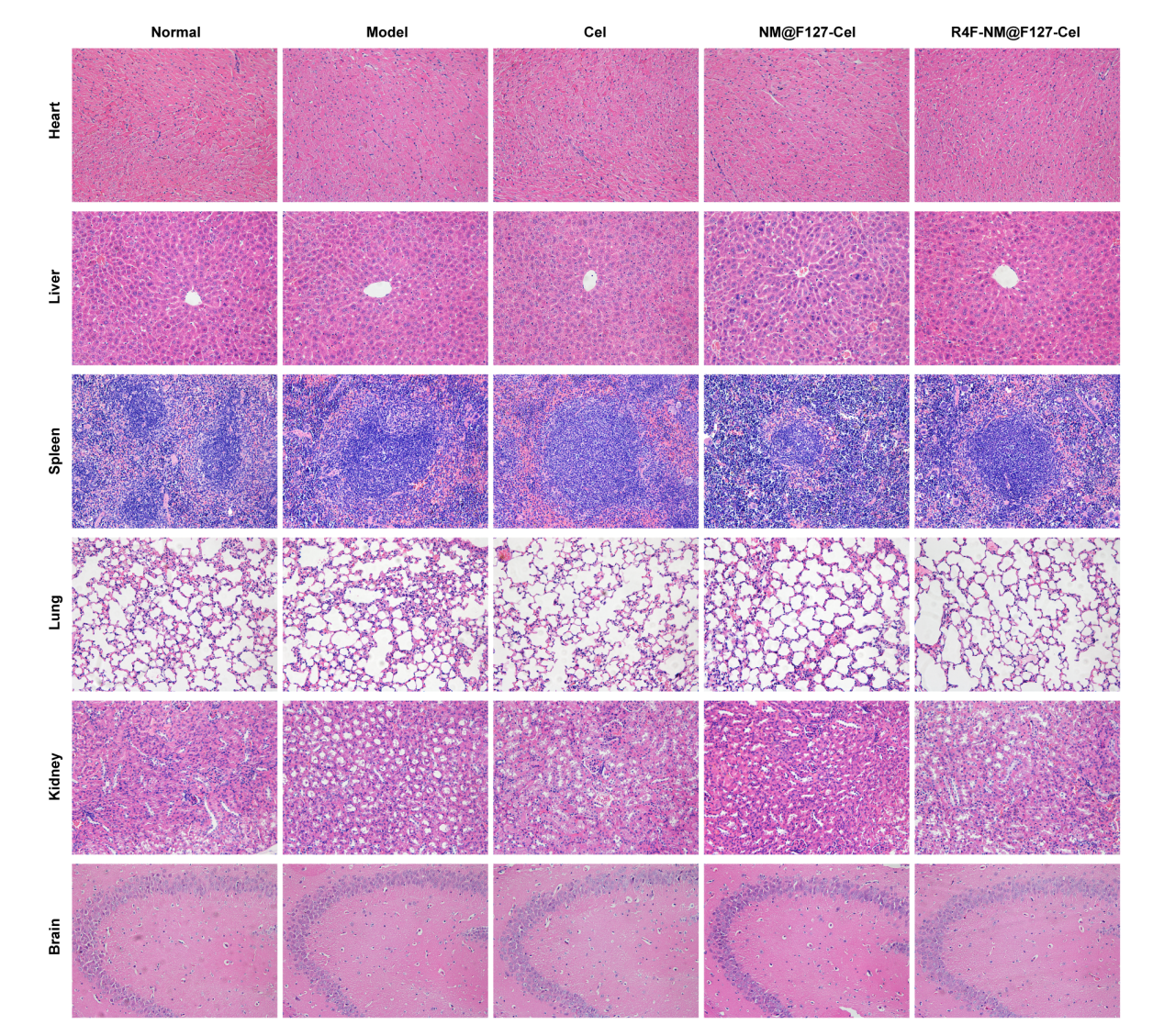


**Fig. S14** H&E staining of heart, liver, spleen, lung, kidney and brain extracted at 45^st^ day after the first immunization (200×).

**
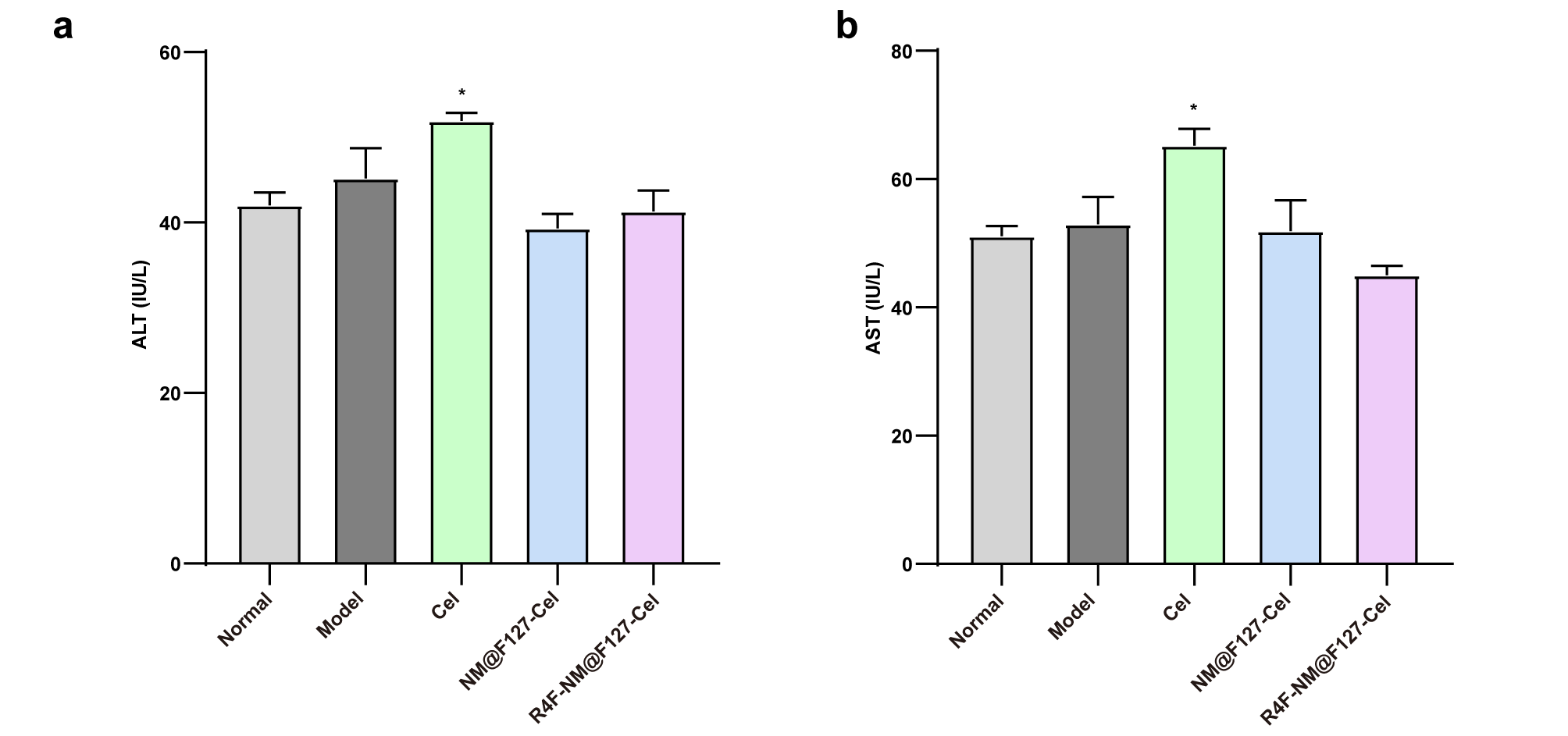
**

**Fig. S15** Levels of ALT (**a**) and AST (**b**) in serum (n = 3). All the treatment groups were compared to normal group. *p < 0.05.

**Supplementary Table**

| **Gene** | **Forward primer (5′ →3′)** | **Reverse primer (5′ →3′)** |
| --- | --- | --- |
| Mouse β-actin | GGCTGTATTCCCCTCCATCG | CCAGTTGGTAACAATGCCATGT |
| Mouse iNOS | AATCTTGGAGCGAGTTGTGG | CAGGAAGTAGGTGAGGGCTTG |
| Mouse TNF-α | CCCTCACACTCAGATCATCTTCT | GCTACGACGTGGGCTACAG |
| Mouse IL-1β | TGGACCTTCCAGGATGAGGACA | GTTCATCTCGGAGCCTGTAGTG |
| Mouse Arg-1 | CTCCAAGCCAAAGTCCTTAGAG | AGGAGCTGTCATTAGGGACATC |
| Mouse IL-10 | GCTCCTAGAGCTGCGGACT | TGTTGTCCAGCTGGTCCTTT |

Table 1. Primer sequences for the amplification.
